# From byproduct to bioactive: genetic profiling, GC-MS, and nutritional analysis of citrus peel extracts for nutraceutical development

**DOI:** 10.3389/fpls.2025.1703245

**Published:** 2025-12-08

**Authors:** Om Prakash, Divya Kushwaha, Kahkashan Perveen, Shaista Arzoo, Saurabh Gangola, Vijay Kumar Juyal, Shifa Khan, Vivekananda

**Affiliations:** 1 Department of Chemistry, Central Council for Research in Ayurvedic Science (CCRAS) - Regional Ayurveda Research Institute, Gwalior, India; 2 Department of Chemistry, Govind Ballabh (G.B.) Pant University, Pantnagar, UK, India; 3 Department of Botany & Microbiology, College of Science, King Saud University, Riyadh, Saudi Arabia; 4 Department of Food Sciences and Nutrition, College of Food Science and Agriculture, King Saud University, Riyadh, Saudi Arabia; 5 Department of Microbiology, Graphic Era (Deemed to be University), Dehradun, India; 6 The Kania School of Management, The University of Scranton, Scranton, PA, United States

**Keywords:** UV-visible spectroscopy, citrus residues, vitamin-C, neutraceuticals, genetic analysis, GC-MS

## Abstract

The phytochemical, nutritional, and therapeutic qualities of citrus species (Rutaceae) make them significant on a global scale. Despite having a wealth of bioactive compounds with significant commercial and nutraceutical potential, citrus by-products, including peels, seeds, and pulp, are routinely discarded in large amounts. In this study, we examine the nutritional composition, elemental diversity, metabolite profile, and genetic variability of citrus species collected from various regions of Uttarakhand, India. Citrus peels are a good source of calcium (up to 104.10mg/100 g), zinc (58.3 mg/100 g), manganese (22.1 mg/100 g) and so on, as well as proteins (12.00 ± 0.11^a^), reducing and non-reducing sugar (136.45 ± .001^a^ and 26.53 ± S1.123^a^), and vitamin C (65.56 ± 0.23^a^), according to elemental and nutritional analyses. On GC analysis, more than 45 bioactive compounds were identified, including rutin, limonene, linoelaidic acid, and palmitic acid. *Citrus jambhiri* (Almora) has the highest (51) phytochemicals, whereas *Citrus sinensis* (Pithoragarh) exhibits the lowest (27) phytochemicals. On genetic analysis, the accessions’ great diversity was further highlighted by a genetic analysis using Simple Sequence Repeat (SSR) markers, which showed grouping patterns related to species and geographic origin. Integrating biochemical, metabolite, and genetic data reveals a strong genotype–environment interaction that influences the nutritional and nutraceutical properties of citrus species. The study provides information that can aid in the development of functional foods, pharmaceutical applications, and crop enhancement methods while highlighting the potential of citrus by-products as sustainable sources of valuable bioactive compounds.

## Graphical Abstract

**Figure:**
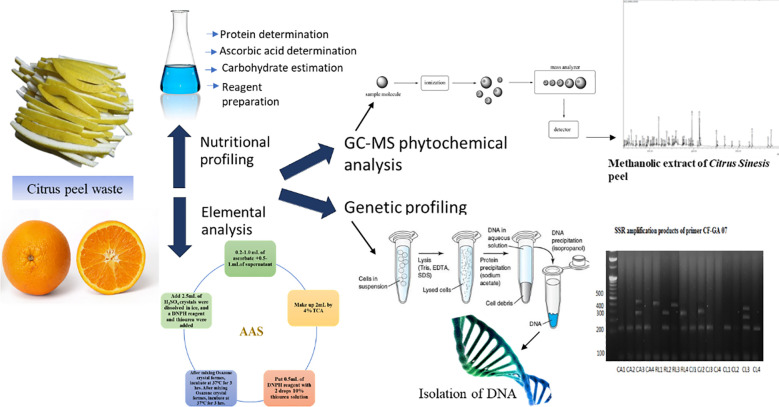
The abstract should ideally be structured according to the IMRaD format (Introduction, Methods, Results and Discussion). Provide a structured abstract if possible. If your article has been copyedited by us, please provide the updated abstract based on this version.

## 1 Introduction

**Figure 1:**
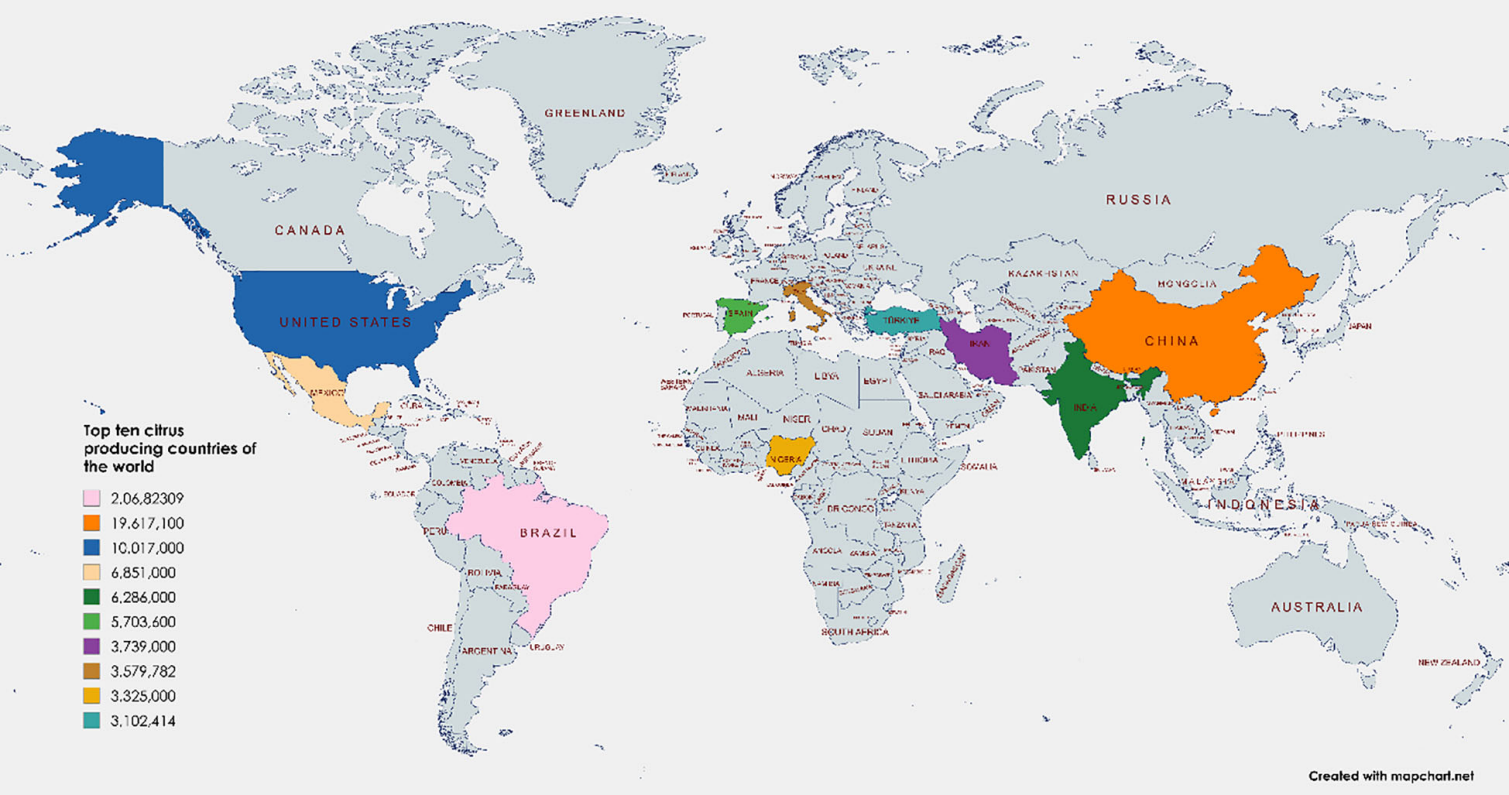
Producing countries of citrus in the whole world.

The possibility of using citrus waste as a source of bioactive chemicals with nutraceutical properties has drawn more attention in recent years. Citrus fruits are widely consumed worldwide due to their invigorating flavor and the numerous nutritional benefits. However, the processing of Citrus fruits results in a good amount of waste, which includes peels, seeds, and pulp. This waste is typically discarded as byproducts (Ezemba et al., 2025). *Citrus L*., as it is scientifically known, belongs to the Rutaceae family, and it is widely cultivated in tropical and subtropical regions, mostly in the Mediterranean and South Asian countries. Citrus is native to South China, northeast India, and Myanmar. The northeast Indian hills and valleys are home to the genuinely untamed citrus populations. They generate about 100 million tons every year. One of the most common fruit crops worldwide is citrus (Citrus spp.), with an estimated yearly yield of 124.24 million tons as shown in **Figure 1**. Citrus is a broad family that includes famous hybrids such as pomelos (*Citrus maxima L*.), grapefruit (*Citrus paradisi L*.), tangerines/mandarins (*Citrus reticulata L.*), sweet oranges (*Citrus sinensis L.*), lemons (*Citrus limon L.*), and limes (*Citrus aurantifolia L.*). According to phytochemical and nutritional assessments, a variety of useful chemicals are found in citrus peels, which consist of Vitamins C, A, and E, minerals, pectin (Murador et al., 2019; Nuzzo et al., 2021), limonoids (Long et al., 2021), flavonoids, carotenoids (El-Kersh et al., 2021; Costanzo et al., 2020), and essential oils (Murador et al., 2019). Citrus peels also contain several additional bioactive substances that have good antioxidant (Long et al., 2021) and health-promoting potential (Mahato et al., 2018; Liu et al., 2021; Gogoi et al., 2021; Abdelghffar et al., 2021). These substances could be used in the pharma industries, food manufacturing, cosmetics, and agricultural sectors (Hou et al., 2019).Apart from this, the pharmaceutical industry might potentially make use of citrus trash. Citrus trash can be used for non-food purposes as a substrate to make packaging materials, activated carbon, bio-fuel, bio-fertilizer, and bio-adsorbent (Sharma et al., 2017)Additionally, because it contains valuable elements like essential oils and has a distinct scent, it is suitable for use in cosmetic items (Pinto et al., 2021).

Utilizing plant leftovers can lower the risk of conditions linked to metabolic syndrome, including cancer, diabetes, cardiovascular disease, and neurological illnesses. Flavonoids, carotenoids, limonoids, terpenes, and other bioactive substances are abundant in *Citrus Lemon* (Rawat et al., 2015). We can take into consideration polyphenols, carotenoids, and essential oils (EOs) as examples of the biologically active compounds (BAC) present in citrus by-products. As per citrus consumed as fresh fruits or goods, the phytochemicals have antimutagenic, antioxidant, anti-carcinogenic (D’Amore et al., 2024), anti-inflammatory, and anti-ageing properties that benefit people’s health (Lv et al., 2015; Prakash et al., 2020).

The neurological system and cardiovascular health are also enhanced by these phytochemicals (Saini et al., 2022). Phytochemicals found in citrus fruits may have antioxidant qualities via strengthening the immune system, increasing the action of liver-protective enzymes, and blocking lipids to stop DNA damage (Ke et al., 2015). It is noteworthy that citrus skin, which is frequently thrown away, has a higher total concentration of polyphenols than the actual peeled fruit Abakpa, G. O., & Adenaike, O. (2021); (Kumar et al., 2019). Although EOs are found in many fruit portions, the peel is particularly noteworthy as a natural source of volatile compounds that have recently piqued the scientific community’s interest. Isoterpenoids, monoterpenes, and sesquiterpenes make up the majority of essential oils (EOs), which are combinations of many chemicals. These BAC provide many aromatic plants with their fragrance (Londoño-Londoño et al., 2010; Rossi et al., 2011) and can be utilized in the production of natural antimicrobials, food flavorings, medicines, and personal care products (Yu et al., 2011). Numerous studies show that vitamin C and B derivatives are widely distributed throughout the citrus species and are especially useful in treating and preventing infectious diseases. Vitamin C helps restructure connective tissue, has antioxidant qualities, and enhances the absorption of iron. The body’s defenses against viruses are strengthened when vitamin C-rich foods are consumed. The antioxidant qualities of citrus bioactive chemicals, including flavonoids, carotenoids, terpenes, and limonoids, might lessen diseases linked to oxidative stress. They can therefore be utilized to treat conditions like cancer, obesity, atherosclerosis, inflammatory illnesses, and neurodegenerative diseases (Lingaraju et al., 2025).

Planning for conservation and breeding crops both depend on an understanding of the genetic diversity of wild plant genetic resources. A number of DNA markers, such as ISSR (inter-simple sequence repeats), RAPD (random amplified polymorphic DNA), AFLP (amplified fragment length polymorphism), and SSR (simple sequence repeats), enable the assessment of genetic diversity within a biological species. Because SSRs are widely distributed throughout the genome, locus-specific, co-dominant, multi-allelic, highly polymorphic, cross-transferable, and analytically inexpensive, they are regarded as perfect DNA markers (Weising et al., 2005; Varshney et al., 2009).

In this literature, we studied on the citrus species collected from the different regions of Uttarakhand. By converting agro-industrial waste into a useful raw material for the food, pharmaceutical, and sustainability industries, this study identifies citrus peel by-products as a promising and underutilized source of nutraceuticals. Citrus wastes contain limonoids, including limonin and nomilin aglycones and limonoid glucosides, including limonin glucoside and nomilic acid glucoside (Kesbiç et al., 2022) which was helpful for the therapeutic purpose and the SSR-based genetic diversity analysis. In addition to revealing important eco-genetic relationships, the results also show how genetic markers influence the biosynthesis of secondary metabolites. This work is new since it emphasizes the value of citrus trash, focuses on both phytochemical richness and genetic variety, and takes an eco-genetic approach to metabolite variance across areas. The study offers new opportunities for breeding tactics, nutraceutical development, and waste-to-wealth innovations in the citrus business by linking various aspects and supporting models of a sustainable bioeconomy.

## 2 Materials and methods

### 2.1 Collection of plant material

The citrus fruit peels were collected from the different regions of Uttarakhand, in which *Citrus limon, Citrus aurentifolia, Citrus sinensis*, and *Citrus jambhiri* were collected from Pithoragarh, Almora, Nainital, and Rudraprayag. The plants were identified by Dr D. S. Rawat, Assistant Professor (Plant taxonomist), Department of Biological Sciences, College of Basic Science and Humanities, Pantnagar. The sampling area, Pithoragarh, lies in the longitudinal and latitudinal coordinates of 29.5829°N and 80.2182°E, Almora lies in 29.5892°N and 79.6467°E, Nanital lies in 29.3924°N and 79.4534°E and at last, Rudraprayag lies in the 30.2850°N and 78.9822°E respectively.

### 2.2 Method of extraction

We collected the Citrus peels from the different regions of Uttarakhand. Then, the citrus peel was shed dried in the room for some days until it was completely dry. Crush the peel into a fine powder form and put it in the Soxhlet method of extraction. In which the powered citrus peel material is placed in the Soxhlet with the appropriate solvent (Methanol) for extraction (Samota et al., 2023). This cycle was repeated 2 to 3 times, and then the solution was filtered through filter paper and collected, which was later dried using a rotary evaporator. And then we got the completely dried methanol extract of citrus peels.

### 2.3 Nutrient profiling

A common technique for measuring sugar with reducing and non-reducing characteristics brought on by the presence of a potential aldehyde or keto group is the colorimetric method using 3, 5-dinitrosalicylic acid (DNS). The method relies on the simultaneous reduction of DNS to 3-amino-5-nitro salicylic acid and the oxidation of functional sugar groups under alkaline and heat conditions, which absorb light at 540 nm.

#### 2.3.1 Determination of protein by spectrophotometric method

In a pestle mortar, 0.5 g of each citrus accession was pulverized with 3 mL of phosphate buffer. These samples were kept overnight. 50 liters of supernatant were adjusted to 250 liters with phosphate buffer, and 3 mL of Bradford dye reagent was added; absorbance was measured at 595 nm in a UV-Visible spectrophotometer against a reagent blank. The protein content was quantified in mg/g, and a standard calibration curve was produced using bovine serum albumin as a reference (Bradford, 1976).

#### 2.3.2 Preparation of reagent

CBBG-250 dye was made by combining 100 mg in 50 mL of ethanol and adding 100 mL (85 percent) orthophosphoric acid, then diluting with double-distilled water to 1 liter (Makarana et al., 2019). The solution was filtered and stored in an amber-colored bottle at 400 degrees Celsius. In phosphate buffer, a 1000 ppm stock solution of BSA was produced (pH 7.6). Various concentrations of BSA were used to create a standard curve.

#### 2.3.3 Estimation of total carbohydrate

100 mg of each sample was thoroughly crushed with 2–3 mL of 5% TCA and 10 mL of 45% ethanol to precipitate the polysaccharides. The tubes were left in the cold overnight and then centrifuged for 10 minutes at 4000 rpm. The dry precipitate was dissolved in 2 mL of 1N NaOH to determine the total carbohydrate content. A sample of up to 1 mL of water was prepared, followed by the addition of 5 mL of concentrated sulfuric acid and 5% phenol. After standing at room temperature for ten minutes, the optical density of the mixture was measured at 490 nm using a spectrophotometer, and a standard curve was created with different D-glucose concentrations. The DNS method was employed to quantify total reducing sugars. After adding 2 mL of 3,5-dinitrosalicylic acid (DNS) to the sample, it was heated for five minutes in a water bath, producing a reddish-brown compound. The concentration of the colored complex was measured at absorbance 540 nm using a spectrophotometer, and a standard curve was generated with various D-maltose concentrations.

#### 2.3.4 Determination of ascorbic acid

0.5 and 1.0 mL of the supernatant and 0.2 to 1.0 mL of standard ascorbate were taken, and the volume was increased by 4% TCA to reach 2 mL. Two drops of a 10% thiourea solution were added to each tube after 0.5 mL of DNPH reagent. Osazone crystals were produced by combining the ingredients and incubating them for three hours at 37 °C. which is dissolved in 2.5 milliliters of cold sulfuric acid. The blank alone was then treated with DNPH reagent and thiourea. After the tubes were cooled in ice, the absorbance was measured at 540 nm in a spectrophotometer. The ascorbate content in the samples was calculated and expressed as milligrams per gram of sample.

### 2.4 Elemental analysis by atomic absorption spectroscopy

AAS (Electronic Corporation of India, Model No. 4141) was used in the determination of the micronutrient (Fe, Zn, Mn, Cu, Na, Ca, Ni, and Co) content of the samples and the total Mg and Ca content was determined using a complexometric approach (Mohammed and Hamza, 2008). We took 0.2-1.0mL of standard ascorbate and 0.5mL - 1.0mL of supernatant. By increasing the volume up to 2.0 mL by 4% of TCA concentration. All of the tubes received 0.5mL of DNPH reagent, followed by 2 drops of thiourea solution (10%). Osazone crystals formed after mixing the materials and incubating them at 37 °C for 3 hours. In 2.5 mL of 85% sulfuric acid, the crystals were dissolved in cold water. After adding sulphuric acid to the blank, the DNPH reagent and thiourea were added and cooled in ice. In a spectrophotometer, the absorbance of the tubes was measured at 540nm. The ascorbate concentration in the samples was determined and given in mg/kg of sample.


$$\text{Element \%}=\text{Reading }\left(\text{ppm}\right)\frac{50\text{mL}}{0.5\text{G}}\frac{1}{100}$$

### 2.5 GC-MS analysis of the methanolic extract of citrus species

For the GC-MS analysis, we use the instrument to identify the essential oils composition in the Methanolic extract. GC**/**MS analysis was carried out using GCMS-QP 2010 (Shumadzu Scientific) Plus equipment at in Advanced Instrument Research Facility, Jawaharlal Nehru University (JNU), New Delhi, with the following conditions: column temperature: 60°C, injection temperature:260°C, Injection mode: split, Flow control: Linear, Pressure: 76.7kPa, Flow rate should be 64.7ml/min, column flow should be in 1.21 ml/min and Purge flow: 3.0 ml/min. The constituents of citrus peel were identified by matching their mass spectra with those in NIST-MS, FFNSC Wiley Library, and comparing with literature reports and GC retention indices (Adams and Sparkman, 2007).

### 2.6 Genetic profiling of citrus accessions

The genetic study was performed in the Department of Microbiology, CBSH, Govind Bhallabh Pant University of Agriculture and Technology, Pantnagar. Instrument used for the gel electrophoresis: Gel Doc ™ XR+System by Bio-Rad Laboratories (USA). Five SSR (Simple sequence repeat) primers were used to investigate the genetic variability among the population of the hurb plant *citrus*, distributed in different parts of Uttarakhand in India. Total genomic DNA from young leaves was extracted using the CTAB (Cetyl Trimethyl Ammonium Bromide) procedure (Southern, 1975; Velichko et al., 2018; Williams et al., 1990) with some modifications.

The quantity and purity of total genomic DNA were checked using a Genesys UV-spectrophotometer of PowerPac ™ Basic Power Study by Bio-Rad Laboratories (USA) by calculating the optical density (OD) at 260 nm and 280 nm. The relation between the concentration of DNA and the optical density is given by:


$$Conc.\;of\;DNA\;\left(µg/mL\right)=\frac{OD_{260}\times 50\times dilution\;factor}{1000}$$

The ratio of OD_260/280_ indicates the presence of RNA or protein contamination in the preparation. The optimum value for the best DNA preparation is 1.8. The ratio<1.8 indicates the presence of protein, and the ratio >1.8 indicates the RNA contamination in the samples.

## 3 Result and discussion

Various parameters have been used to analyze the nutritional and elemental profiling of the Citrus species collected from the different parts of Uttarakhand. And the variation of the element and nutrition percentage has been identified by following the mentioned parameters, and the chemical profiling of the plants has been identified by the GC-MS, which shows the content variation, also identifies the genetic variability, which differs from region to region.

### 3.1 Determination of proteins by spectrophotometric method

**Figure 2:**
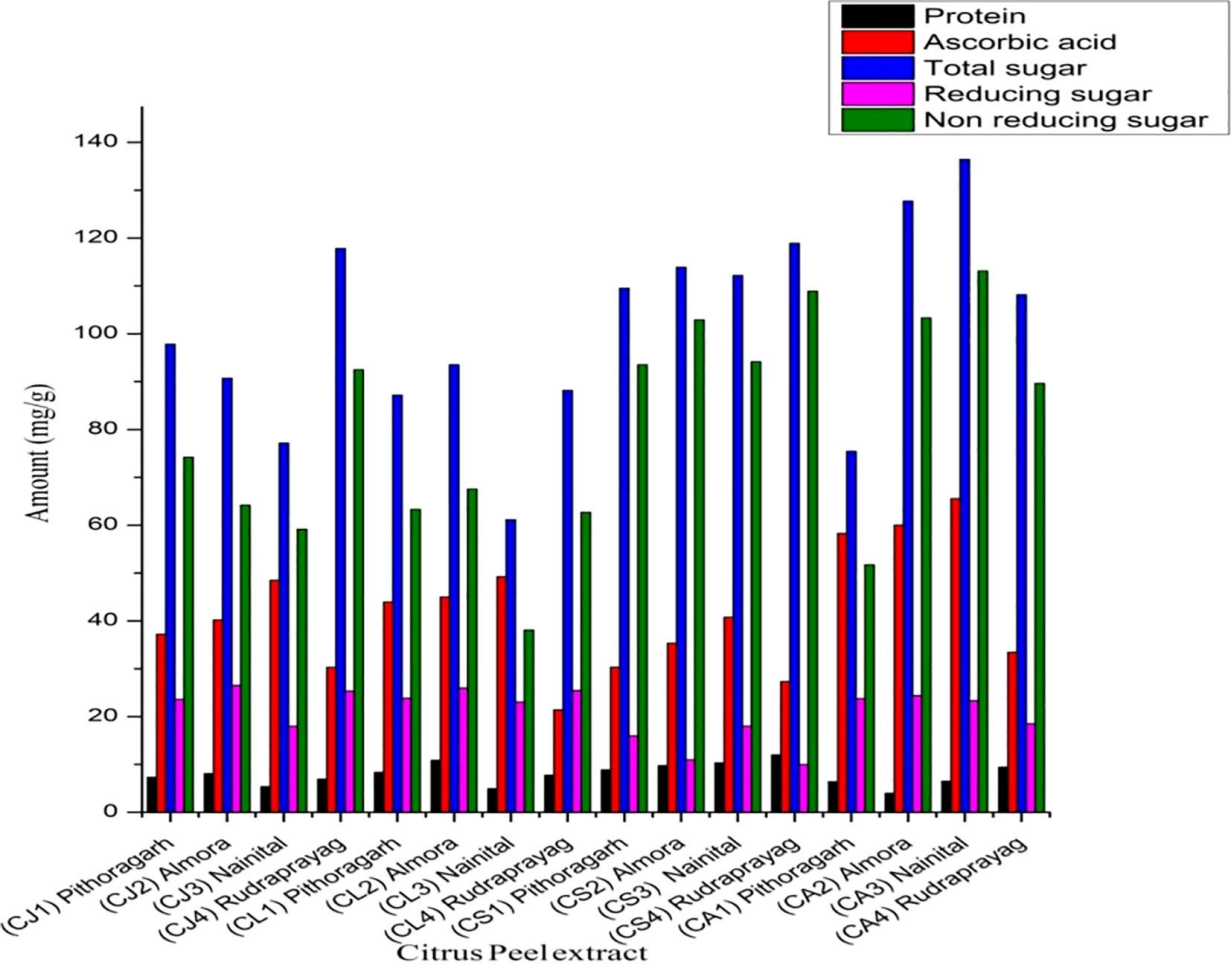
Nutritional profiling of citrus peel extract collected from the Uttarakhand region.

Protein is a nitrogenous macromolecule made up of amino acids linked together with peptide linkage. It was the first to describe a dye in a colorimetric reagent for the detection and quantification of total proteins. All protein assays are performed using Coomassie dye binding assays, which are the quickest and easiest to perform (Nehad et al., 2025). In **Figure 2**, you can see the dye’s spectrum change from reddish-brown to blue (absorbance maximum at 465 nm to 610 nm). *Citrus sinensis* from Rudraprayag had the highest protein level (12.000 ± 0.11), while *Citrus aurentifolia* from Almora had the lowest protein content (4.000 ± .33). These samples differed considerably (p<0.05) and ranged from 12.000 ± .11 to 4.000 ± 0.33. The above finding revealed that the protein content of all citrus accessions varies greatly (Bradford, 1976). discovered a similar result.

#### 3.1.2 Ascorbic acid content

Ascorbic acid, another name for vitamin C, is an essential vitamin needed for both human and animal existence. Water-soluble vitamin C provides many health advantages, such as preventing cancer and scurvy, relieving cold symptoms, promoting the production of collagen, and promoting wound healing (Stern et al., 2004).

The ascorbic acid content of all citrus accessions of the Uttarakhand region differed significantly from each other (p<0.05) and varied from 65.56 ± 0.23 to 21.45 ± 0.45 mg/g, and its calibration curve is depicted in **Figure 1B**. The maximum ascorbic acid content was recorded in *Citrus aurentifolia* from Nainital (65.56 ± 0.23), while the least amount of ascorbic acid was found in *Citrus limon* Rudraprayag (21.45 ± 0.45) as shown **Table 1A** in **Supplementary Data** (Ahmad et al., 2012) reported 4.23% ascorbic acid content in citrus peel extract.

**Table 1:** Showing the abundance of phytomolecules in each species, with the percentage of extract.

S. no.	Plant material (peel of methanolic extract	Location	No. of chemical constituents in the methanolic extract out of 100	Percentage in total extract
1	*Citrus jambhiri*	Pithoragarh	39	92.90%
2	*Citrus jambhiri*	Almora	51	78.80%
3	*Citrus jambhiri*	Nanital	34	61.75%
4	*Citrus jambhiri*	Rudraprayag	44	81.97%
5	*Citrus limon*	Pithoragarh	41	79.49%
6	*Citrus limon*	Almora	38	71.85%
7	*Citrus limon*	Nanital	41	65.43%
8	*Citrus limon*	Rudraprayag	42	83.37%
9	*Citrus sinensis*	Pithoragarh	27	77.01%
10	*Citrus sinensis*	Almora	46	66.37%
11	*Citrus sinensis*	Nanital	39	84.54%
12	*Citrus sinensis*	Rudraprayag	39	97.45%
13	*Citrus aurentifolia*	Pithoragarh	41	92.41%
14	*Citrus aurentifolia*	Almora	47	57.29%
15	*Citrus aurentifolia*	Nanital	32	67.85%
16	*Citrus aurentifolia*	Rudraprayag	42	93.37%

#### 3.1.2 Total sugar content

In the present work, quantitative estimation of total carbohydrate present in different accessions of Citrus was observed using the phenol sulphuric acid method. The amount of total sugar varied from 136.45 ± .001 to 61.18 ± .031mg/g and was significantly higher (p<0.05) as depicted in **Supplementary Data**, **Figure 1B**. The maximum amount of total sugar content was recorded in *Citrus aurentifolia* collected from Nainital (136.45 ± .001), while the least amount of total sugar was found in *Citrus limon* collected from Nainital (61.18 ± .031) as depicted in the **Supplementary File** Similarly, the sugar content in *Citrus aurentifolia* is reported by (Kumar et al., 2019) to be higher in *Citrus jambhiri*.

#### 3.1.3 Total reducing and non- reducing sugars

A common technique for measuring sugars with reducing and non-reducing characteristics caused by aldehyde or keto groups is the colorimetric 3,5-dinitrosalicylic acid (DNS) method. Under alkaline and hot conditions, the idea is based on the reduction of DNS to 3-amino-5-nitro salicylic acid, which absorbs at 540 nm, and the oxidation of sugar functional groups (Ting, 1956). To improve color intensity, phenol is frequently added, and the alkaline NaOH medium guarantees that DNS and reducing sugars have the right redox interaction (Jimenez Moreno et al., 2025). The reducing sugar content of Uttarakhand citrus accessions was determined to range from 26.53 ± 1.123 mg/g to 10.00 ± 0.014 mg/g using this approach. *Citrus sinensis* from Rudraprayag had the lowest value (10.00 ± 0.014 mg/g), whereas *Citrus jambhiri* from Almora had the highest value (26.53 ± 1.123 mg/g). These values were significantly different (p< 0.05). Similarly, there was a large range in the non-reducing sugar concentration, from 113.12 ± 0.421 mg/g to 38.11 ± 0.021 mg/g. Nainital’s *Citrus aurentifolia* had the highest concentration (113.12 ± 0.421 mg/g), while the citrus lemon had the lowest concentration (38.11 ± 0.021 mg/g). The biochemical variety of citrus accessions in Uttarakhand is highlighted by these findings, which show variations in sugar metabolism between species and geographical areas (Hazarika et al., 2025).

### 3.2 Elemental analysis

Elemental analysis was carried out by atomic absorption spectroscopy for the determination of Cu, Fe, Mn, Zn, Co, Ni, Na, and Ca, which have been reported in mg/100g. A detailed analysis of data is depicted in **Table 1B** in the **Supplementary File**. The iron content varies between 114μg/gm and 220 μg/gm. Iron is an essential mineral because it is a component of enzymes involved in energy production, protein metabolism, nucleotide metabolism, and the synthesis of proteins and neurotransmitters, among other processes. It is a component of hemoglobin and myoglobin, and hence plays a role in the body’s oxygen delivery (Soetan et al., 2010). Citrus is also rich in vitamin C. Supplementing with iron capsules is insufficient to treat iron deficiency because iron absorption is also a crucial factor. It’s been discovered that vitamin C boosts iron’s mucosal absorption capability. *Citrus sinensis* peel from Nainital (4.4 mg/100g) provides a link between copper and iron metabolism and mediates the release of iron from ferritin and hemosiderin in **Table 1B**. An excessive amount of copper causes hypertension, sporadic fever, and coma. Investigations on copper content.

**Figure 3:**
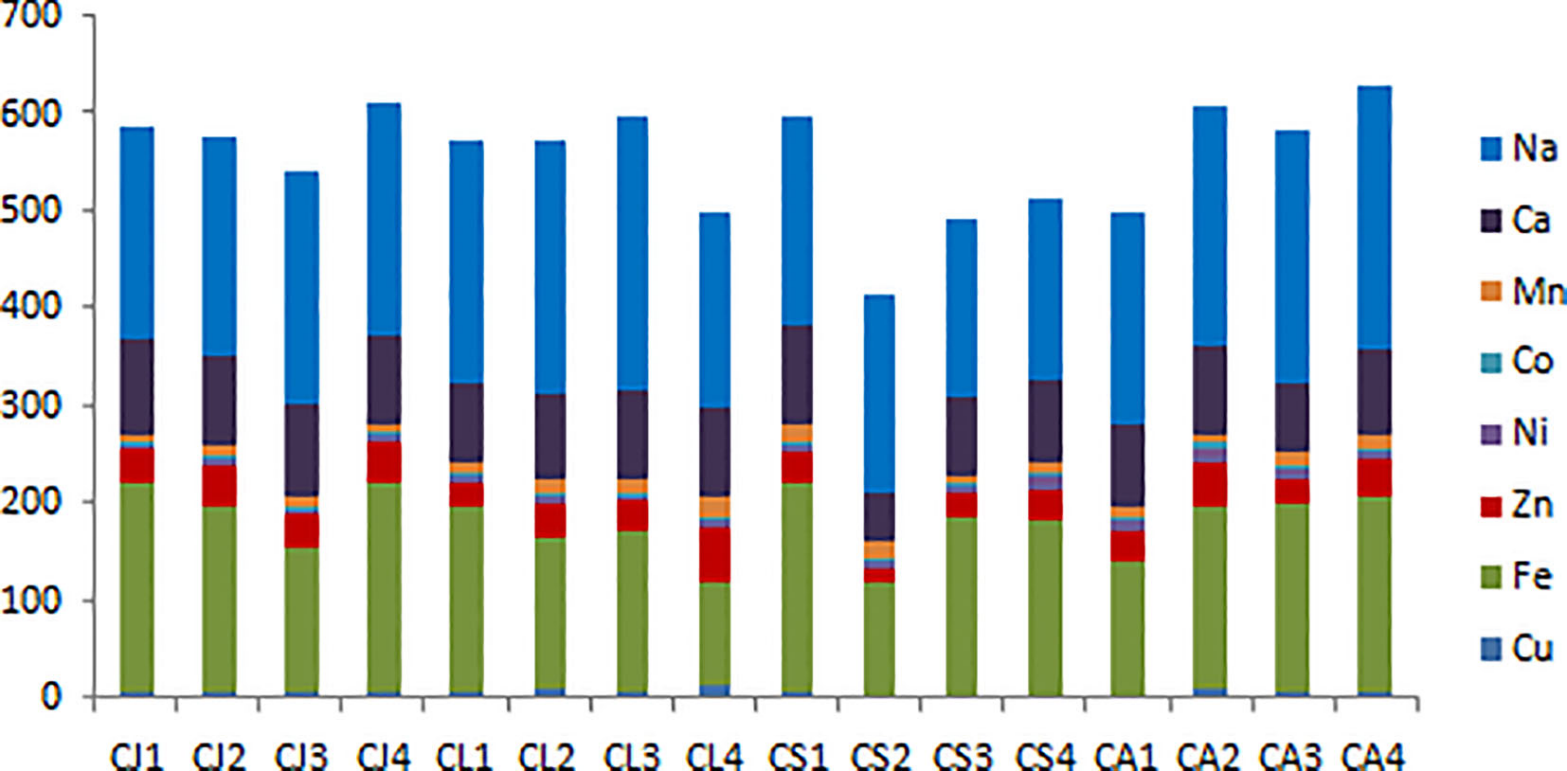
Element profiling of citrus peel extract from the Uttarakhand region (mg/100g), in which the x-axis denotes the CJ, *Citrus jambhiri*; CL, *Citrus lemon*; CS, *Citrus sinensis*; CA, *Citrus aurentifolia*; collected from the 1. Pithoragarh, 2. Almora, 3. Nainital,4. Rudrprayag.

Citrus accessions from Uttarakhand showed significant species and location-specific diversity in their mineral profiles. *Citrus sinensis* (Almora) had the lowest iron concentration and *Citrus jambhiri* (Pithoragarh) the highest, with an iron content ranging from 114 to 220 μg/g. Copper values in citrus lemons (Rudraprayag) peaked at 4.4–12.7 mg/100g. With the highest concentrations found in *Citrus aurentifolia* (Rudraprayag), the manganese content ranged from 7 to 22.2 mg/100g. *Citrus limon* (Rudraprayag) had the highest zinc content, with a range of 16.7–58.3 mg/100g. 184–271.7 mg/100g of sodium were found, with *Citrus aurentifolia* (Rudraprayag) having the highest values. The cobalt level of *Citrus aurentifolia* (Almora) was the highest, ranging from 0.9 to 6.8 mg/100g. Between 5 and 14.6 mg/100g, nickel levels peaked in *Citrus sinensis* (Rudraprayag) comparison of the content well explained in the Bar chart of **Figure 3**. With *Citrus sinensis* (Pithoragarh) having the highest calcium levels, the range was 50.8–104.1 mg/100g. When compared to those from Almora and Nainital, accessions from Rudraprayag and Pithoragarh generally showed higher mineral contents, showing nutritional variety particular to both species and regions.

### 3.3 Chemical profiling of citrus accessions by GC-MS analysis

Gas chromatogram of different species of citrus peels’ methanolic extracts collected from the Kumaun and Garhwal region. While the identification of individual compounds present in the methanolic extract of different species having the same district and different species having different districts of citrus peels was compared with the mass spectra present in various libraries (Gupta et al., 2014),. Although during the study we found that the *Citrus jambhiri* (Almora) contains the highest number (51) of phytochemicals, and the lowest (27) in the *Citrus sinensis* (Pithoragarh). *Citrus jambhiri* (Pithoragarh) exhibits a total of 39 phytomolecules. *Citrus jambhiri* (Nanital) contains a total of 34 phytomolecules, and *C. jambhiri* (Rudraprayag) contains a total of 44 phytomolecules. whereas *Citrus limon* (Pithodagarh) contains 41 no. of phytomolecules, *C. limon* (Almora) contains 38 no. of phytomolecules, *C. limon* (Nanital) exhibits a total 41 no. of phytomolecules, and *C. limon* (Rudraprayag) contains a total 42 phytochemicals. In *Citrus sinensis* (Pithoragarh) contains 27 phytochemicals, *C. sinensis* (Almora) contains a total 46 no. of phytochemicals, and *C. sinensis* (Nanital) and (Pithoragarh) exhibit the same 39 no. of phytomolecules. And in *Citrus aurentifolia* (Pithoragarh), 41 phytomolecules were found, *C. aurentifolia* (Almora) showed 47 no. of phytochemicals, *C. aurentifolia* (Nanital), and C. aurentifolia contains 42 phytochemicals. Based on phytochemical profiling of GC-MS found that some molecules like- Rutin, Linoleic acid, Palmitic acid, Myristic acid, Limonene, and octonic acid are mostly found in every species of the Citrus in almost every region. Only the abundance of the phytomolecules may vary from region to region of the species. some more compounds like Resorcinol, Geranial, Linalool, Cytidine, Steric acid, and so on, which can vary plant to plant with the ecological variation and environmental factors. Detailed analysis of the phytoconstituents with the chromatogram and the availability is shown in the **Supplementary Data**. Table no. 3.3.1 to 3.3.14.

Based on the above discussion on the supplementary GC data file, the compound o-butylisourea was only found in *C.jambhiri* Pithoragarh, same as Linalyl acetate only found in *C. aurentifolia* (Nanital). Myristic acid, Octadecanoic acid, methyl ester, Linoelaidic acid, Γ-sitosterol, and Monoterpenes were found in *C. jambhiri* (Pithoragarh) and *C.sinensis*. (Rudraprayag).Eicosanoic acid was found in *C. sinensis* (Almora).

### 3.4 Genetic profiling of citrus accessions

**Figure 4:**
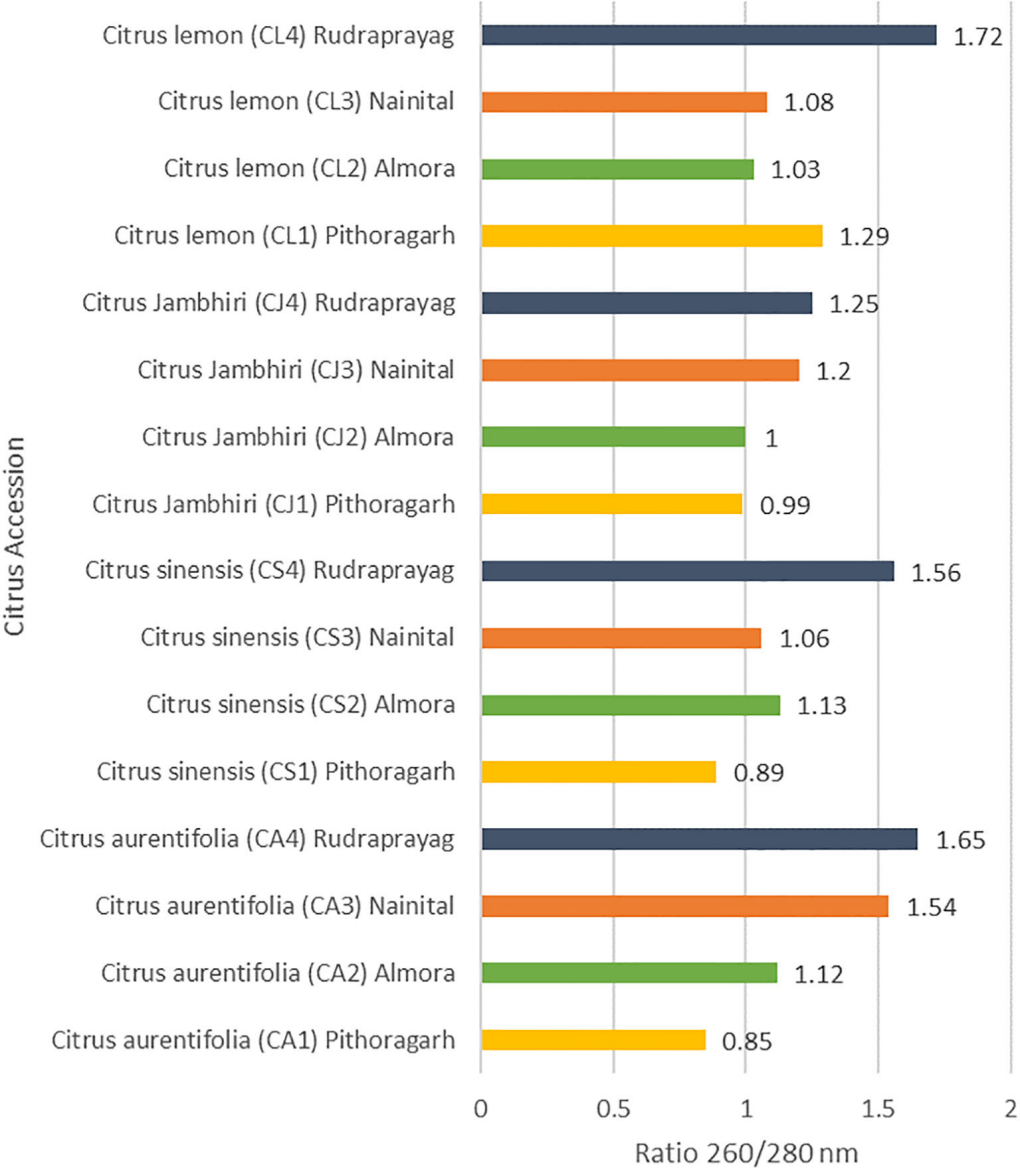
Optical density of DNA isolated from citrus accessions.

SSR marker analysis was carried out in order to study the genetic diversity of different citrus accessions from the Kumaun and Garhwal region of Uttarakhand. Sixteen citrus accessions were compared against five SSR (Simple Sequence Repeat) primers. Primers varied greatly in their ability to resolve variability in some of the samples. Genetic diversity among all accessions was explained through **Table 2** and **Figure 4**.

**Table 2:** Amplified products of 16 different citrus accessions.

S.No	Marker	No of alleles	No of polymorphic bands	Allele size range (bp)	PIC value
1.	CF-GT 02	20	6	100–320 bp	0.70
2.	CF-TA 03	24	5	100–450 bp	0.79
3.	CF-AT 13	33	6	100–450 bp	0.81
4.	CMS 07	17	4	100–300 bp	0.76
5.	CF-GA 07	21	6	100–400 bp	0.76
Average value			5.0		0.76


$$PIC=\;1-\sum \;{\left(Pi\right)}^{2}$$

The purity of DNA calculated for samples from the 260/280 ratio of optical density ranges between 0.85 to 1.72. Maximum polymorphic bands are present in the case of CF-GT 02. The PIC value comes out between 0.58 to 0.75 with an average value of 0.62 detailed explaination in **Figure 4**. PIC value is a good measure of heterozygosity, associated with a high degree of polymorphism (Ramadugu et al., 2015). Primer CF-GA07 produced the maximum, while primer CF-GA07–07 produced the minimum number of bands, respectively. The genetic similarities among the citrus accessions were calculated through Jaccard’s coefficients using NTYSYS-PC software version 2.01. Similarity coefficient values ranged between 0.20 to 1.0 (**Table 3**). The highest similarity coefficients were found between CS1 and CJ4 accession; on the other hand, the lowest similarity coefficient, 0.25, was found between CS4 and CJ2.

**Table 3:** Genetic similarity matrix of sixteen accessions of citrus based on SSR analysis.

	CA1	CA2	CA3	CA4	CL1	CL2	CL3	CL4	CS1	CS2	CS3	CS4	CJ1	CJ2	CJ3	CJ4
CA1	1	0.462	0.5	0.455	0.5	0.25	0.5	0.167	0.462	0.278	0.357	0.143	0.267	0.077	0.412	0.357
CA2		1	0.353	0.5	0.25	0.462	0.417	0.176	0.5	0.294	0.286	0.154	0.385	0.182	0.353	0.5
CA3			1	0.25	0.667	0.412	0.467	0.562	0.438	0.588	0.533	0.25	0.278	0.125	0.75	0.438
CA4				1	0.133	0.333	0.273	0.133	0.667	0.267	0.364	0.091	0.364	0.111	0.176	0.5
CL1					1	0.235	0.462	0.467	0.333	0.5	0.538	0.308	0.25	0.071	0.562	0.333
CL2						1	0.286	0.5	0.357	0.533	0.188	0.231	0.357	0.4	0.333	0.462
CL3							1	0.267	0.417	0.5	0.308	0.167	0.308	0.2	0.375	0.545
CL4								1	0.333	0.714	0.538	0.417	0.333	0.25	0.471	0.429
CS1									1	0.467	0.636	0.25	0.385	0.083	0.278	0.8
CS2										1	0.467	0.357	0.467	0.308	0.5	0.571
CS3											1	0.25	0.286	0	0.438	0.5
CS4												1	0.25	0.25	0.176	0.25
CJ1													1	0.3	0.438	0.5
CJ2														1	0.125	0.182
CJ3															1	0.278
CJ4																1

#### 3.4.1 Hetrozygosity

**Figure 5:**
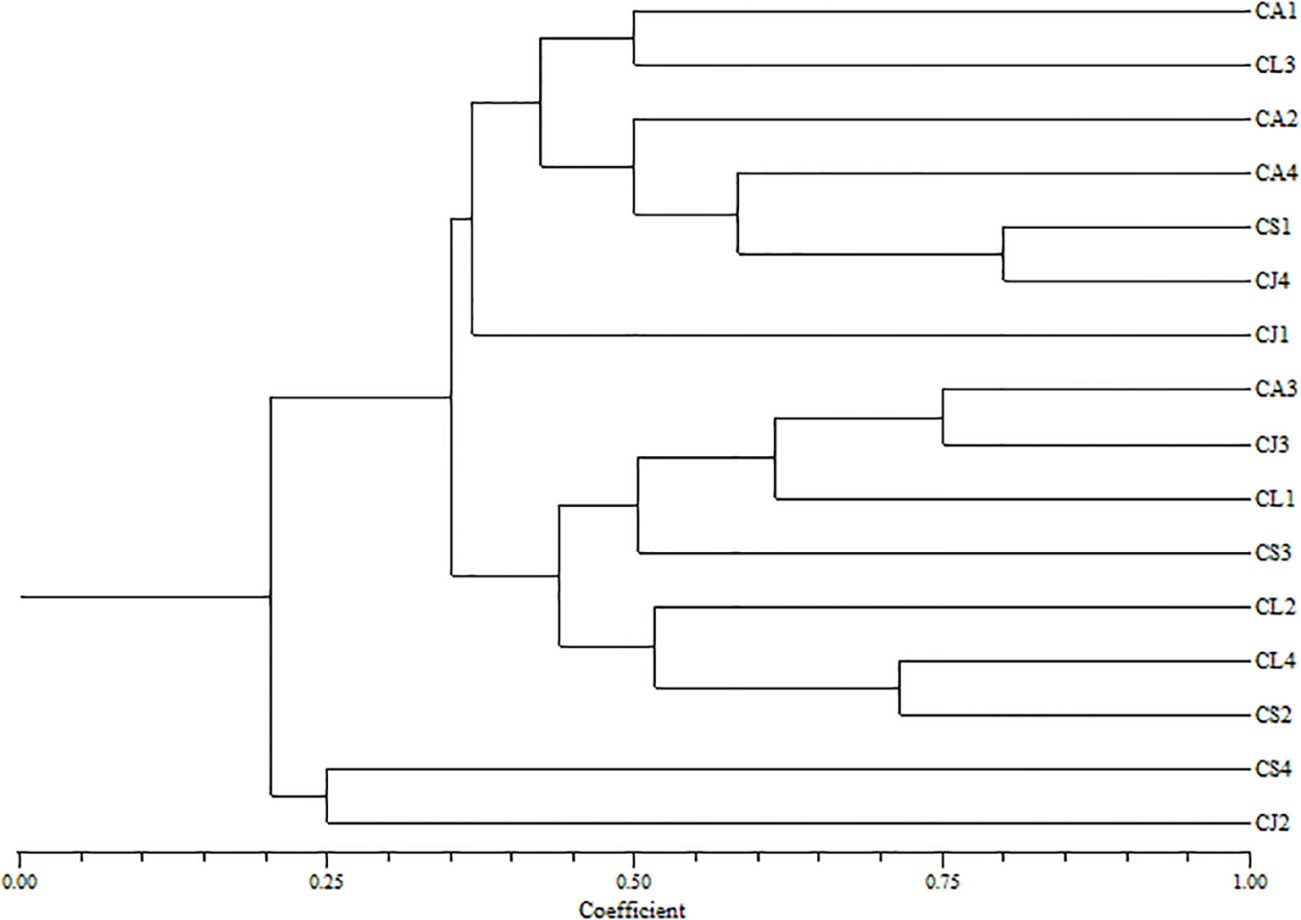
UPGMA cluster analysis showing the diversity relationship among citrus accessions using SSR primers.

Primer A produced maximum bands while primers B and C produced minimum bands, respectively. The genetic similarities among the citrus accessions were calculated through Jaccard’s coefficients using NTYSYS-pc software 2.01. Similarity coefficient values ranged between 0.10 to 0.83 in **Figure 5**. The highest similarity coefficient values were found between CA1 and CA2, CJ1 and CJ2 accessions. On the other hand, the lowest similarity coefficient of 0.10 was found between CJ4 and CL1. The heterozygosity observed with the microsatellite data included in the study ranged from 0 to 86.96% **Figure 5**. Citrus from the same district had a lower level of heterozygosity than other districts. In citrus species, the heterozygosity ranged from 0 to 52%. However, according to previous reports (Barkley et al., 2006), certain citrons showed a high level of heterozygosity. *Citrus jambhiri* had 52.17% heterozygous SSR markers, *Citrus limon* had 43.38% heterozygosity, *Citrus aurentifolia* and *Citrus sinensis* had a high percentage of heterozygosity, 82.61%.

#### 3.4.2 Gene Analysis

Amplification of SSR was conducted using a total of 5 primer pairs distributed throughout the citrus genome. Data from 5 primer pairs that were able to discriminate the citron accessions were utilized for the study. In total, 61 putative alleles were detected, with an average of 7 alleles per locus. Six unique citron alleles were observed in accessions from microsatellite loci CF-AT13 and CF-TA03, which had a maximum of 8 alleles each; locus CF-ACA01 had 4 alleles, the lowest number recorded for this dataset (**Table 3**). The amplicon sizes ranged from 126 to 400 bp. The discriminating power of each microsatellite marker was determined by calculating the PIC value (Nagy et al., 2012). For the primers used in this study, the PIC value ranged from 0.23 (JK-CA23) to 0.75 (CF-GT02) (**Table 2**).

**Figure 6:**
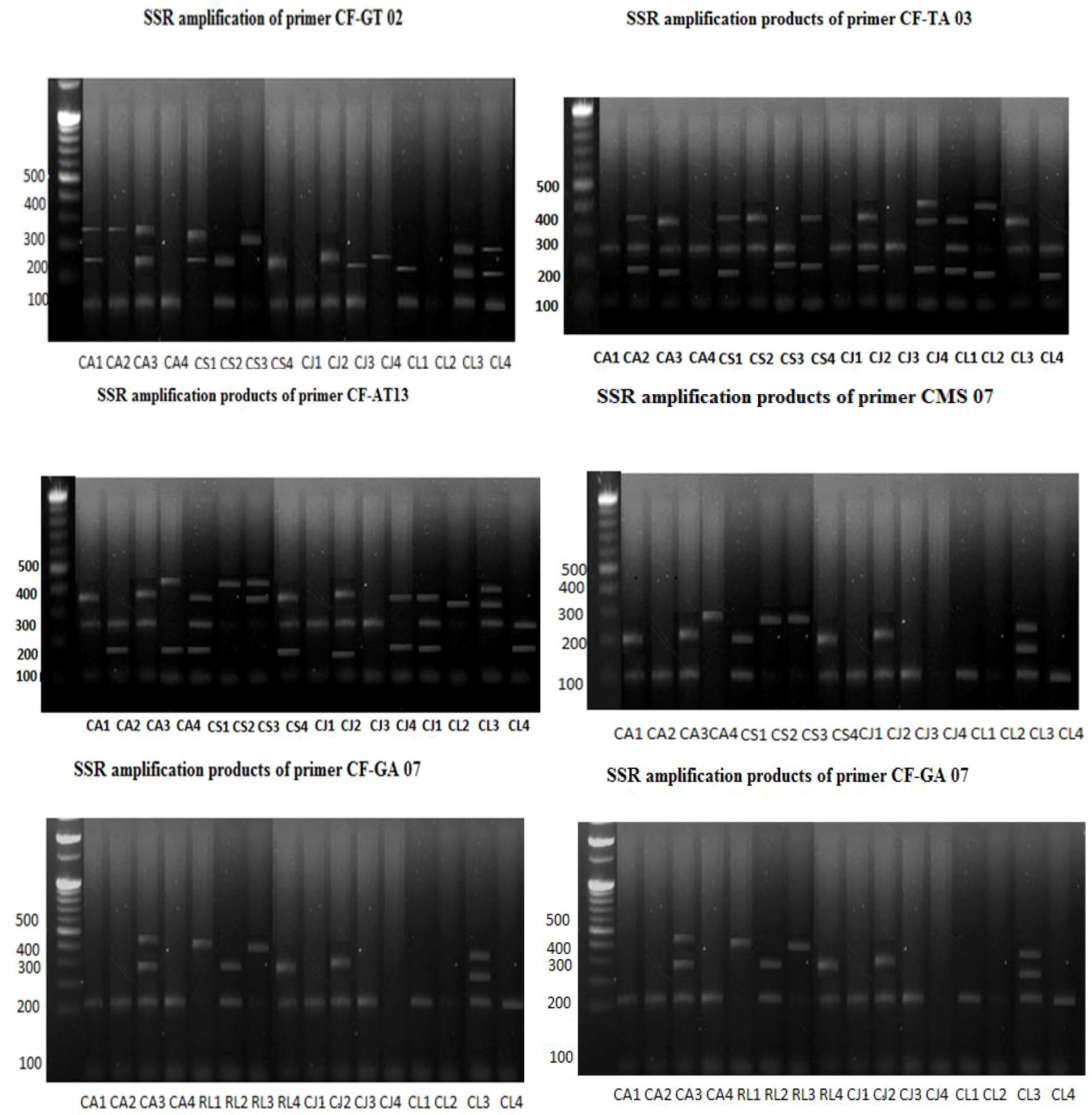
Genetic diversity of citrus using SSR markers with all sixteen accessions (CA1-CL4) collected from Pithoragarh, Almora, Nainital, and Rudraprayag.

The UPGMA clustering method is based on (Nagy et al., 2012). Classify all sixteen accessions into main groups (I and II) with four clusters. The first main groups are further subdivided into three clusters, the subgroups (IA and IIB). IIB is again subdivided into three subgroups. CA1,CL3,CA2,CA4,CS1,CJ4,CJ1 while second sub cluster exhibited single accession CJ1, third cluster exhibited CA3,CJ3,CL1,CS3,CL2,CL4,CS2 and fourth sub cluster exhibited CS4,CJ2.First cluster of subgroups IIB contained CS1 and CJ4 with 80% similarity, and found to be 50% similar with CA1, CL3, CA2, CS3, and CL2 exhibited similarity and found to be 60% similar with CA4 and CL1, the subgroups IIB deviated further again CL4 and CS2 have 72% exhibited similarity, while CA3 and CJ3 exhibited 75% similarity as shown in **Figure 6**. Likewise, the main group of (II) CS4 and CJ2 showed similarity at 25%.

The UPGMA clustering method based on (de la Parra et al., 2024) classifies all the 16 citrus species accessions into two main groups (1 and 2) with five clusters (**Figure 5**). The first main group further deviates into two subgroups, 1A and 1 B. The IA subgroups again deviated into three clusters. The first cluster comprises CJ1, CJ2, CJ3, CJ4, second cluster exhibited CS1, CS2. The first group has single accessions of citrus species.

The genetic similarity coefficient is an important tool for extrapolating the genetic relationship between citrus accessions. A similarity coefficient of 0.83 was observed between CJ1 and CJ2, which provides evidence that these accessions from the same parent CA1 citrus accessions possess the minimum similarity coefficient, resulting in maximum variation among all studied citrus accessions. Finally, the results concluded that the citrus collected from Pithoragarh and Almora are genetically the same. Citrus samples collected from Nainital and Rudraprayag are genetically similar. From this, we found that citrus accessions that are regionally close have maximum similarity as compared to those accessions that are far apart.

Also, environmental factors play a great role in the genetic makeup of plants. Our findings confirm that (Bhalerao et al., 2003) reported the genetic diversity of citrus using SSR markers.

### 3.5 Genetic diversity and chemical characterization of citrus accessions

In order to assess the diversity and nutraceutical potential of citrus accessions gathered from four districts of Uttarakhand (Pithoragarh, Almora, Nainital, and Rudraprayag), this work combines GC–MS metabolite profiling with SSR-based genetic analysis. Significant differences in the quantity and percentage of metabolites were found by GC-MS analysis between species and geographical areas. The chemical variety of *Citrus jambhiri* was lowest in Nainital (34 chemicals, 61.75%) and highest in Almora (51 compounds, 78.80%).

*C. lemon* and *C. sinensis* showed similar trends, with extract percentages ranging from 65.43% to 97.45% and many chemicals ranging from 38 to 46. Among the metabolites*, C. sinensis* from Rudraprayag had the highest abundance (97.45%). The range of *C. aurentifolia* compounds was vast, from 32 in Nainital to 47 in Almora, with the highest percentage (93.37%) found in Rudraprayag. These findings demonstrate how metabolite richness is affected by both environmental factors and genetic origin. Whereas similarity values ranging from 0.10 to 0.83 were found using SSR-based genetic analysis, suggesting significant variation between accessions. Significant genetic divergence was indicated by the lowest similarity (0.10) between CJ4 and CL1, while high similarity was discovered between CJ1–CJ2 and CA1–CA2, both of which were from close regions. Similar metabolite yields were frequently shown by genetically similar accessions, such as *C. jambhiri* from Almora and Pithoragarh (51 and 39 compounds). This implies that the production of phytochemicals may be influenced by genetic similarities. However, instances of divergence, in which the amount of extracts varied among genetically similar accessions, highlight the significance of metabolic plasticity and environmental modification.

An average of seven putative alleles per locus was amplified by the gene analysis utilizing five pairs of SSR primers. Strong marker discriminating strength was demonstrated by the polymorphic information content (PIC) values, which varied from 0.23-0.75. Genes controlling the synthesis of metabolites may be represented by loci CF-AT13 and CF-TA03, each of which has eight alleles. 16 accessions were divided into two main groups and numerous subgroups using UPGMA clustering. CJ1–CJ2 and CA1–CA2 are two examples of accessions from the same districts that often congregated together, demonstrating how common ecological constraints influence both genetic and phytochemical features. While Nainital and Rudraprayag formed separate groups reflecting their varied metabolite abundances, Pithoragarh and Almora accessions showed tight clustering and comparable metabolite profiles. The comparison of GC-MS and SSR data reveals a robust interaction between environment and genetics in determining nutraceutical potential. While moderately heterozygous species like *C. jambhiri* (52.17%) and *C. limon* (43.38%) maintained consistent yields across districts, genetically divergent accessions like CJ4 and CL1 showed both low similarity and significant differences in metabolites, making them dependable for nutraceutical applications. *C. sin*ensis and *C. aurentifolia*, on the other hand, showed more metabolite variability due to their higher heterozygosity (82.61%), which makes them ideal genetic resources for breeding programs that aim to increase phytochemical richness. Interestingly, extract percentages were consistently greater in Rudraprayag accessions, highlighting the part environmental adaptation plays in metabolite accumulation.

## 4 Limitation and toxicity of citrus peel

According to the above study, the sample size was limited to four citrus species collected from the same selected regions of Uttarakhand, which does not show the complete variation of ecologically and genetically because we cannot control the seasonal variation, maturity stages and post-harvest handling. On GC-MS and SSR analysis, it provides details in chemical and genetic characterization, and the study can include transcriptomics and metabolomics, which can give deeper insights into the gene-metabolite interactions.

If we talk about the toxicity of the citrus peel extract, which was common for the insecticidal activities in a research of the toxicity of citrus extract against adult Anopheles mosquitoes at L50. The peels showed the lowest toxicity of 43.04, while the extract from *C. limonum* seeds had the maximum toxicity of 96.10. Peel extracts from *C. sinensis* had the highest mortality rate at 60 minutes of L50 (39.56), while those from *C. aurantifolia* had the lowest mortality rate (22.05). as increasing the concentration of the citrus extract increases the toxicity (Simon-Oke and Akeju, 2019). Extracts from all citrus species contained saponins, which are incredibly toxic since they hemolyze blood cells and can harm cattle. Besides irritating mucous membranes, they have an unpleasant and bitter taste. Terpenoid was present in every extract. When administered physically, terpenes irritate; when ingested internally, they have an effect similar to that of gastrointestinal system irritants. Among the triterpenes are cardiac glycosides, steroids, and sterols that have cytotoxic, anti-inflammatory, sedative, or insecticidal properties (Kar, 2007).

## 5 Conclusion

Citrus fruit peels, which are typically disposed of as industrial trash, are now recognized by this study as significant bioresources with exceptional nutritional and nutraceutical potential. It offers a thorough grasp of the connection between genetic composition, ecological variation, and metabolite abundance across four citrus species (*Citrus jambhiri, Citrus limon, Citrus sinensis*, and *Citrus aurentifolia*) by combining nutritional composition, GC–MS metabolite profiling, and SSR-based genetic diversity analysis. Citrus peels are appropriate for nutraceutical and pharmaceutical uses since they are high in vital elements like Calcium, Zinc, Manganese, Proteins, Carbohydrates, Phytochemicals, and Vitamin C, according to the research. Significant regional and interspecific differences in nutrient and metabolite levels were noted, underscoring the robust impact of environmental and genetic variables on metabolite diversity. *Citrus aurentifolia* from Almora had the lowest Protein and phytochemical content, whereas *Citrus lemon* from Rudraprayag had the greatest. Likewise, regional differences in vitamin C and carbohydrate content highlight how genotype–environment interactions influence nutritional profiles.

While SSR-based genetic diversity analysis identified unique genetic variations among the citrus species, GC-MS profiling revealed a variety of bioactive compounds with antioxidant and therapeutic properties. Collectively, these results show that the biosynthesis of useful secondary metabolites is driven by both ecological and genetic variables.

All things considered, this work connects biodiversity, sustainability, and bioeconomic value by providing a fresh eco-genetic viewpoint on the use of citrus peels. Encouraging the transformation of citrus waste into useful nutraceutical resources, it supports waste-to-wealth initiatives and advances a sustainable, circular bioeconomy.

## Data Availability

The raw data supporting the conclusions of this article will be made available by the authors, without undue reservation.
