# Supplementary material for: From byproduct to bioactive: genetic profiling, GC-MS, and nutritional analysis of citrus peel extracts for nutraceutical development
Source: Front Plant Sci. 2025 Dec 8;16:1703245. doi: 10.3389/fpls.2025.1703245 (PMC12719522; doi:10.3389/fpls.2025.1703245)
Supplement: Supplementary file 1 [file DataSheet1.doc]

**Supplementary data of Citrus species**

**Abbreviation:**

| ***Citrus aurentifolia*( Pithoragarh)** | **CA1** | |
| --- | --- | --- |
| ***Citrus aurentifolia* (Almora)** | **CA2** | |
| ***Citrus aurentifolia* (Nanital)** | **CA3** | |
| ***Citrus aurentifolia* (Rudraprayag)** | **CA4** | |
| ***Citrus sinensis* ( Pithoragarh)** | **CS1** | |
| ***Citrus sinensis* (Almora)** | **CS2** | |
| ***Citrus sinensis* (Nanital)** | **CS3** | |
| ***Citrus sinensis* (Rudraprayag)** | | **CS4** |
| ***Citrus lemon* ( Pithoragarh)** | **CL1** | |
| ***Citrus lemon* (Almora)** | **CL2** | |
| ***Citrus lemon* (Nanital)** | **CL3** | |
| ***Citrus lemon* (Rudraprayag)** | **CL4** | |
| ***Citrus jambhiri* ( Pithoragarh)** | **CJ1** | |
| ***Citrus jambhiri* (Almora)** | **CJ2** | |
| ***Citrus jambhiri* (Nanital)** | **CJ3** | |
| ***Citrus jambhiri* (Rudraprayag)** | **CJ4** | |

**3.1 Nutritional profiling of the Citrus species**

A common technique for measuring sugar with reducing and non-reducing characteristics brought on by the presence of a potential aldehyde or keto group is the colorimetric method using 3, 5-dinitrosalicylic acid (DNS). The method relies on the simultaneous reduction of DNS to 3-amino-5-nitro salicylic acid and the oxidation of functional sugar groups under alkaline and heat conditions, which absorb light at 540 nm. The DNS alkaline medium (NaOH) is necessary for the redox reaction between DNS and the reducing sugar. In the Uttarakhand region, the reducing sugar content ranged from 26.53±1.123 mg/g to 10.00±0.014 mg/g. *Citrus jambhiri* from Almora had the highest total sugar content (26.53±1.123), while *Citrus sinensis* Rudraprayag had the lowest (10.00±0.014), which was significantly higher (p<0.05). The decreasing sugar content of several citrus peel extract accessions is shown in Table1A

**Table 1A: Nutritional profiling of citrus peel extract collected from Uttarakhand**

| **S.No.** | **Altitude** | **Citrus accessions** | **Ascorbic acid (mg/g)** | **Total sugar (mg/g)** | **Total reducing sugar (mg/g)** | **Total non-reducing sugar (mg/g)** | **Protein(mg/g)** |
| --- | --- | --- | --- | --- | --- | --- | --- |
|  |
| 1 | **1514 m** | (CJ1) Pithoragarh | 37.24±1.06^j^ | 97.81±.001^i^ | 23.60±0.120^h^ | 74.21±0.421^i^ | 7.34±0.03^j^ |
| 2 | **1642 m** | (CJ2) Almora | 40.21±1.01^i^ | 90.72±.001^k^ | **26.53±S1.123^a^** | 64.19±0.422^k^ | 8.09±0.32^h^ |
| 3 | **2084 m** | (CJ3) Nainital | 48.50±0.93^e^ | 77.18±.002^n^ | 18.00±0.122^l^ | 59.84±0.321^n^ | 5.38±0.045^n^ |
| 4 | **895 m** | (CJ4) Rudraprayag | 30.30±0.12^n^ | 117.81±.005^d^ | 25.33±0.121^e^ | 92.48±0.421^g^ | 6.96±0.09^k^ |
| 5 | **1514 m** | (CL1) Pithoragarh | 43.96±0.68^g^ | 87.18±.008^m^ | 23.86±0.10^f^ | 63.31±0.22^l^ | 8.37±0.010^g^ |
| 6 | **1642 m** | (CL2) Almora | 45.01±0.32^f^ | 93.54±.021^j^ | 26.00±1.130^b^ | 67.54±0121^j^ | 10.90±0.05^b^ |
| 7 | **2084 m** | (CL3) Nainital | 49.23±1.56^d^ | **61.18±.031^p^** | 23.06±0.140^i^ | **38.11±0.021^p^** | 4.96±0.010^o^ |
| 8 | **895 m** | (CL4) Rudraprayag | **21.45±0.45^p^** | 88.18±.001^l^ | 25.46±0.120^c^ | 62.71±0.221^m^ | 7.76±0.40^i^ |
| 9 | **1514 m** | (CS1) Pithoragarh | 30.34±0.21^m^ | 109.54±.002^g^ | 16.00±0.100^m^ | 93.67±0.421^f^ | 8.90±0.03^f^ |
| 10 | **1642 m** | (CS2) Almora | 35.34±0.23^k^ | 113.90±.009^e^ | 11.00±0.020^n^ | 102.10±0.521^d^ | 9.76±0.04^d^ |
| 11 | **2084 m** | (CS3) Nainital | 40.78±0.21^h^ | 112.19±.001^f^ | 18.03±0.120^k^ | 94.64±0.021^e^ | 10.34±0.12^c^ |
| 12 | **895 m** | (CS4) Rudraprayag | 27.34±0.21^o^ | 118.90±.001^c^ | **10.00±0.014^o^** | 108.17±033^b^ | **12.00±0.11^a^** |
| 13 | **1514 m** | (CA1) Pithoragarh | 58.30±0.93^c^ | 75.45±.023^o^ | 23.73±0.01^g^ | 51.72±0.421^o^ | 6.40±0.001^m^ |
| 14 | **1642 m** | (CA2) Almora | 60.01±0.91^b^ | 127.72±.045^b^ | 24.40±1.016^d^ | 103.32±0.21^c^ | **4.00±0.33^p^** |
| 15 | **2084 m** | (CA3) Nainital | **65.56±0.23^a^** | **136.45±.001^a^** | 23.33±0.017^f^ | **113.12±0.421^a^** | 6.50±0.44^l^ |
| 16 | **895 m** | (CA4) Rudraprayag | 33.45±0.24^l^ | 108.18±.001^h^ | 18.53±0.018^j^ | 89.64±0.021^h^ | 9.43±0.00^e^ |

Values are means of three replicates ±SD. Within a column, means values followed by the same letters are not significantly different at P ≤ 0.05. The superscript used in the table (**a to p**) is according to the decreasing order of the content in a single test, like in ascorbic acid citrus Accession, (CA3) Nanital **65.56±0.23^a^ has** the highest content, and (CL4) Rudraprayag has the lowest content of Ascorbic acid **21.45±0.45^p^.**


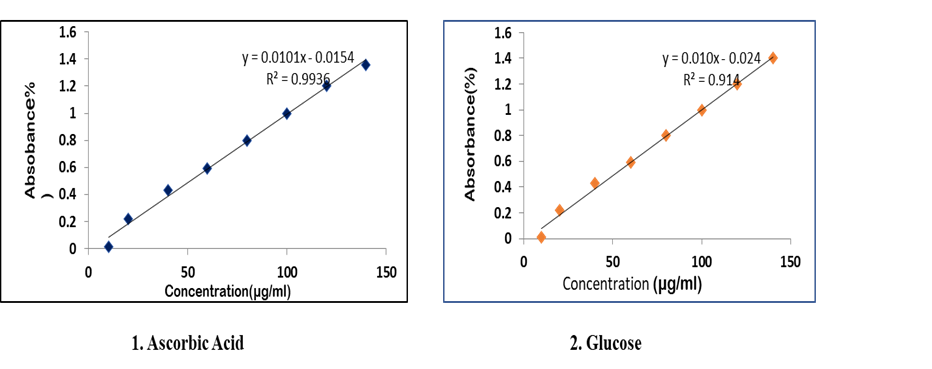


**Figure 1B: Calibration curve of A. Ascorbic acid B. Glucose**

**3.2 Elemental analysis**

Atomic absorption spectroscopy was used to do elemental analysis, and the amounts of Cu, Fe, Mn, Zn, Co, Ni, Na, and Ca were reported in milligrams per hundred grams. Fig. 1.3 and Table 2.3. Minerals are the building blocks of bodily tissues and play a role in muscular contraction, gas transport, fluid management, and the preservation of the acid-base balance. The estimation of micronutrients reveals the variation in micronutrient levels among citrus genotypes. While the levels of sodium and calcium were not statistically different (p<0.05), the levels of iron, zinc, manganese, calcium, copper, sodium, cobalt, and nickel varied significantly between genotypes. One essential micronutrient is calcium. In addition to its role in blood coagulation, it is necessary for the healthy operation of the heart, muscles, and nerves. A lack of it might result in osteoporosis. The order of macro and micro-necessary elements in Uttarakhand's citrus accession was Na>Fe>Ca>Zn>Mn>Cu>Ni>Co.

**Table 1B: Element profiling of citrus peel extract from Uttarakhand region (mg/100g)**

| **S.No** | **Citrus accessions** | **Altitude**  **(meters)** | **Cu** | **Fe** | **Zn** | **Ni** | **Co** | **Mn** | **Ca** | **Na** |
| --- | --- | --- | --- | --- | --- | --- | --- | --- | --- | --- |
| 1 | (CJ1) Pithoragarh | **1514 m** | 5.40 | 216.10 | 34.80 | 7.90 | 0.90 | 7.00 | 97.30 | 219.70 |
| 2 | (CJ2) Almora | **1642 m** | 5.90 | 192.10 | 40.10 | 12.10 | 1.30 | 7.30 | 91.70 | 226.30 |
| 3 | (CJ3) Nainital | **2084 m** | 7.50 | 147.70 | 34.70 | 5.30 | 2.70 | 9.50 | 96.70 | 237.00 |
| 4 | (CJ4) Rudraprayag | **895 m** | 6.10 | 213.80 | 42.20 | 11.40 | 1.80 | 7.50 | 90.40 | 239.50 |
| 5 | (CL1) Pithoragarh | **1514 m** | 7.10 | 188.80 | 24.20 | 8.10 | 2.80 | 12.00 | 81.80 | 250.10 |
| 6 | (CL2) Almora | **1642 m** | 10.10 | 156.00 | 35.30 | 9.10 | 2.00 | 13.70 | 88.30 | 257.40 |
| 7 | (CL3) Nainital | **2084 m** | 8.60 | 162.80 | 33.40 | 6.00 | 1.10 | 17.90 | 91.90 | 279.80 |
| 8 | (CL4) Rudraprayag | **895 m** | 12.70 | 106.30 | 58.30 | 5.00 | 3.40 | 22.10 | 92.40 | 198.30 |
| 9 | (CS1) Pithoragarh | **1514 m** | 6.30 | 216.80 | 30.40 | 5.10 | 4.10 | 17.80 | 104.10 | 214.60 |
| 10 | (CS2) Almora | **1642 m** | 4.60 | 114.00 | 16.70 | 6.70 | 3.30 | 15.00 | 50.80 | 204.40 |
| 11 | (CS3)Nainital | **2084 m** | 4.40 | 182.50 | 23.10 | 10.80 | 2.10 | 6.80 | 80.30 | 184.00 |
| 12 | (CS4) Rudraprayag | **895 m** | 4.40 | 177.10 | 33.50 | 14.60 | 3.90 | 8.80 | 83.90 | 186.70 |
| 13 | (CA1) Pithoragarh | **1514 m** | 5.00 | 134.20 | 34.20 | 10.70 | 3.80 | 8.60 | 84.70 | 217.50 |
| 14 | (CA2) Almora | **1642 m** | 11.60 | 184.200 | 48.20 | 11.30 | 6.80 | 9.80 | 91.20 | 245.10 |
| 15 | (CA3) Nainital | **2084 m** | 7.10 | 195.100 | 23.90 | 10.50 | 2.20 | 13.50 | 72.30 | 257.80 |
| 16 | (CA4) Rudraprayag | **895 m** | 5.60 | 201.600 | 38.90 | 8.00 | 1.80 | 15.10 | 87.70 | 271.70 |

**3.3 Chemical profiling of Citrus accessions by GC-MS analysis**

We use the instrument to identify the essential oils composition in the Methanolic extract. GC**/**MS analysis was carried out using GCMS-QP 2010 Plus equipment at in Advanced Instrument Research Facility, Jawaharlal Nehru University (JNU), New Delhi, with the following conditions: column temperature: 60℃, injection temperature: 260℃, Injection mode: split, Flow control: Linear, Pressure: 76.7kPa, Flow rate should be 64.7ml/min, column flow should be in 1.21 ml/min and Purge flow: 3.0 ml/min. The constituents of citrus peel were identified by matching their mass spectra with those in NIST-MS, FFNSC Wiley Library, and comparing with literature reports and GC retention indices

**3.3.1 GC-MS analysis of the methanolic extract of citrus species**

The four different species of citrus peels (*Citrus jambhiri, Citrus lemon, Citrus aurentifolia, and Citrus sinensis)* collected from four different districts, three of the Kumaun region and one district of the Garhwal region, and each four different species of citrus from having different districts were subjected to GC-MS analysis. All these species showed significant variation in their phytoconstituents.

GC-MS analysis of ***Citrus jambhiri*** peels of methanolic extracts (Pithoragarh) reveals the presence of 100 constituents, out of which 39 constituents contributing 92.9% of the total extracts were identified **(Table 3.3.1).**

GC-MS analysis of peels of methanolic extracts (Almora) reveals the presence of 100 constituents, out of which 51 constituents contributing 78.8% of the total extracts were identified **(Table 3.3.2).**

GC-MS analysis of ***Citrus jambhiri*** peels of methanolic extracts (Nainital) reveals the presence of 100 constituents, out of which 34 constituents contributing 61.75% of the total extracts were identified **(Table 3.3.3).**

GC-MS analysis of ***Citrus jambhiri*** peels of methanolic extracts (Rudraprayag) reveals the presence of 100 constituents, out of which 44 constituents contributing 81.97% of the total extracts were identified **(Table 3.3.4).**

GC-MS analysis of ***Citrus lemon*** peels of methanolic extracts (Pithoragarh) reveals the presence of 100 constituents, out of which 41 constituents contributing 79.49% of the total extracts were identified **(Table 3.3.5).**

GC-MS analysis of ***Citrus lemon*** peels of methanolic extracts (Almora) reveals the presence of 100 constituents, out of which 38 constituents contributing 71.85% of the total extracts were identified **(Table 3.3.6).**

GC-MS analysis of ***Citrus lemon*** peels of methanolic extracts (Nainital) reveals the presence of 100 constituents out of which 41 constituents contributing 65.43% of the total extracts were identified **(Table 3.3.7).**

GC-MS analysis of ***Citrus lemon*** peels of methanolic extracts (Rudraprayag) reveals the presence of 100 constituents out of which 42 constituents contributing 83.37% of the total extracts were identified **(Table 3.3.8).**

GC-MS analysis of ***Citrus sinensis*** peels of methanolic extracts (Pithoragarh) reveals the presence of 100 constituents, out of which 27 constituents contributing 77.01% of the total extracts were identified **(Table 3.3.9).**

GC-MS analysis of ***Citrus sinensis*** peels of methanolic extracts (Almora) reveals the presence of 100 constituents, out of which 46 constituents contributing 66.37% of the total extracts were identified **(Table 3.3.10).**

GC-MS analysis of ***Citrus sinensis*** peels of methanolic extracts (Nainital) reveals the presence of 100 constituents, out of which 39 constituents contributing 84.54% of the total extracts were identified **(Table 3.3.11).**

GC-MS analysis of ***Citrus sinensis*** peels of methanolic extracts (Rudraprayag) revels the presence of 100 constituents, out of which 39 constituents contributing 97.45% of the total extracts were identified **(Table 3.3.12).**

GC-MS analysis of ***Citrus aurentifolia*** peels of methanolic extracts (Pithoragarh) reveals the presence of 100 constituents, out of which 41 constituents contributing 92.41% of the total extracts were identified **(Table 3.3.13).**

GC-MS analysis of ***Citrus aurentifolia*** peels of methanolic extracts (Almora) reveals the presence of 100 constituents, out of which 47 constituents contributing 57.287% of the total extracts were identified **(Table 3.3.14).**

GC-MS analysis of ***Citrus aurentifolia*** peels of methanolic extracts (Nainital) reveals the presence of 100 constituents, out of which 32 constituents contributing 67.85% of the total extracts were identified **(Table 3.3.15).**

GC-MS analysis of ***Citrus aurentifolia*** peels of methanolic extracts (Rudraprayag) reveals the presence of 100 constituents out of which 42 constituents contributing 93.37% of the total extracts were identified **(Table 3.3.16).**

**Fig 3.3.1: Gas chromatogram of methanolic extract of *Citrus jambhiri* peels, Pithoragarh**

**Table 3.3.1: Chemical composition of *Citrus jambhiri*** collected from Pithoragarh

| **S.No** | **Name** | **Area%** | **R. Time** |
| --- | --- | --- | --- |
| 1 | o-butylisourea | 3.31 | 4.213 |
| 2 | 2,2- Dichlorobutane | 2.38 | 4.28 |
| 3 | Acetonyl acetate | 0.47 | 4.839 |
| 4 | Cyclopent-4-ene-1,3-dione | 0.32 | 5.117 |
| 5 | Dihydroxyacetone | 2.95 | 5.295 |
| 6 | 2-shydroxy-γ-butyrolactone | 3.01 | 6.931 |
| 7 | (+)-Limonene | 1.61 | 7.691 |
| 8 | Cyclopropylmethanol | 1.26 | 8.708 |
| 9 | Octanoic acid | 0.39 | 9.832 |
| 10 | Α-ketoisovaleric acid | 0.28 | 10.433 |
| 11 | Geranial | 3.83 | 10.612 |
| 12 | 2-undecanone | 1.84 | 11.719 |
| 13 | 2-methoxy-4-vinylphenol | 4.29 | 12.024 |
| 14 | Decanoic acid | 1.11 | 12.642 |
| 15 | Linalool | 10.6 | 13.195 |
| 16 | Cytidine | 0.04 | 13.815 |
| 17 | 2-tridecanone | 2.32 | 14.421 |
| 18 | 2-hydroxyisocaproic acid | 7.95 | 14.506 |
| 19 | Undecanoic acid | 0.31 | 15.108 |
| 20 | Dodecanoic acid | 1.31 | 15.225 |
| 21 | L-(+)-lactic acid, trimethylsilyl ester | 0.46 | 15.756 |
| 22 | Hexanoic acid, tms derivative | 5.71 | 15.951 |
| 23 | Dodecanoic acid | 10.31 | 16.278 |
| 24 | Tetradecanoic acid | 5.4 | 17.485 |
| 25 | Myristic acid, | 3.35 | 18.429 |
| 26 | 5-(2,4-dimethoxyphenyl)cyclohexane-1,3-dione | 6.51 | 18.872 |
| 27 | Octadecanoic acid, methyl ester | 0.8 | 19.243 |
| 28 | N-hexadecanoic acid | 0.71 | 19.578 |
| 29 | Palmitic acid | 3.6 | 20.395 |
| 30 | Linoelaidic acid | 4.13 | 21.226 |
| 31 | Cis-11-Eicosenoic acid | 3.64 | 21.282 |
| 32 | Octadecanoic acid | 0.8 | 21.488 |
| 33 | 9,12-(z,z)- octadecadienoic acid | 0.22 | 21.922 |
| 34 | 9-octadecenoic acid | 0.38 | 21.973 |
| 35 | Stearic acid | 0.45 | 22.2 |
| 36 | Rutin | 4.75 | 33.187 |
| 37 | Γ-sitosterol | 2.21 | 36.83 |
| 38 | 3',4',5,6,7,8-hexamethoxyflavone | 3.43 | 37.496 |
| 39 | Monoterpenes | 0.46 | 44.21 |
|  | **Total** | **92.9.9%** |  |

**Fig 3.3.2: Gas chromatogram of methanolic extract of *Citrus jambhiri* peels (Almora)**

**Table 3.3.2: Chemical composition of *Citrus jambhiri*** collected from Almora

| **S.No** | **Name** | **Area%** | **R.Time** |
| --- | --- | --- | --- |
| 1 | 1,3-cyclopentenedione | 4.21 | 5.11 |
| 2 | Dihydroxyacetone | 1.64 | 5.321 |
| 3 | Phenol | 0.89 | 6.758 |
| 4 | 2-Hydroxy-γ-butyrolactone | 1.64 | 6.938 |
| 5 | Octanal | 1 | 7.202 |
| 6 | Pentanal | 0.85 | 8.705 |
| 7 | 1-Buty l(dimethyl) silyloxypropane | 0.25 | 9.175 |
| 8 | 2-acetyl-2-hydroxy γ butyrolactone | 2.18 | 9.289 |
| 9 | Azulene | 1.16 | 10.261 |
| 10 | Decanal | 2.64 | 10.463 |
| 11 | 2,3-dihydro-benzofuran | 0.8 | 10.611 |
| 12 | 2-undecanone | 0.2 | 11.718 |
| 13 | 2-Furanacetic acid | 3.2 | 11.95 |
| 14 | 2-Methoxy-4-vinylphenol | 3.77 | 12.024 |
| 15 | Resorcinol | 0.26 | 12.425 |
| 16 | 4-hydroxybenzaldehyde | 1.03 | 12.861 |
| 17 | 2-phenylisopropanol | 0.24 | 13.13 |
| 18 | 1-chloro-4-methoxy Benzene | 0.24 | 13.192 |
| 19 | Sucrose | 11.39 | 13.65 |
| 20 | Decanoic acid | 0.12 | 13.903 |
| 21 | 1-chloro-Hexadecane | 0.27 | 14.15 |
| 22 | 1-chloro-4-methoxy-Benzene | 0.65 | 14.313 |
| 23 | (+) Limonene | 0.67 | 14.403 |
| 24 | Farnesol | 0.7 | 14.503 |
| 25 | 2-Oxabicyclo[2.2.2]octan-6-ol, 1,3,3-trimethyl acetate | 1.45 | 14.606 |
| 26 | Aceburic acid | 0.17 | 15.02 |
| 27 | 2-Ketoisocaproic acid | 0.51 | 15.116 |
| 28 | Decanoic acid | 1 | 15.186 |
| 29 | Octadecanoic acid | 0.64 | 15.268 |
| 30 | 1,2-Benzenedicarboxylic acid, | 0.13 | 15.5 |
| 31 | Hexanoic acid | 0.24 | 15.757 |
| 32 | 2,E-Prostaglandin | 0.31 | 16.508 |
| 33 | 2-Hydroxyisocaproic acid, trimethylsilyl ester | 0.3 | 17.118 |
| 34 | Tetradecanoic acid | 0.57 | 17.477 |
| 35 | 2,3-dimethyl-4-quinolinone | 0.15 | 18.242 |
| 36 | Myristic acid | 0.88 | 18.429 |
| 37 | Eicosenoic acid | 1.02 | 19.242 |
| 38 | N-Hexadecanoic acid | 0.54 | 19.578 |
| 39 | Hexadecanoic acid, trimethylsilyl ester | 4.69 | 20.395 |
| 40 | Petasitene | 0.2 | 21.067 |
| 41 | Eicosenoic acid, methyl ester | 0.06 | 21.175 |
| 42 | Linoelaidic acid | 5.84 | 21.23 |
| 43 | 7-(z)-tetradecenal | 2.03 | 21.284 |
| 44 | Octadecanoic acid | 0.7 | 21.489 |
| 45 | Linoelaidic acid | 0.26 | 21.921 |
| 46 | 9-Octadecenoic acid | 0.29 | 21.975 |
| 47 | Stearic acid | 1.09 | 22.188 |
| 48 | Heneicosane | 0.38 | 23.885 |
| 49 | Rutin | 6.84 | 25.542 |
| 50 | 4',5,6,7,8-pentamethoxyflavone | 3.24 | 33.191 |
| 51 | 3',4',5,6,7,8-hexamethoxyflavone | 5.27 | 37.502 |
|  | **Total** | **78.8%** |  |


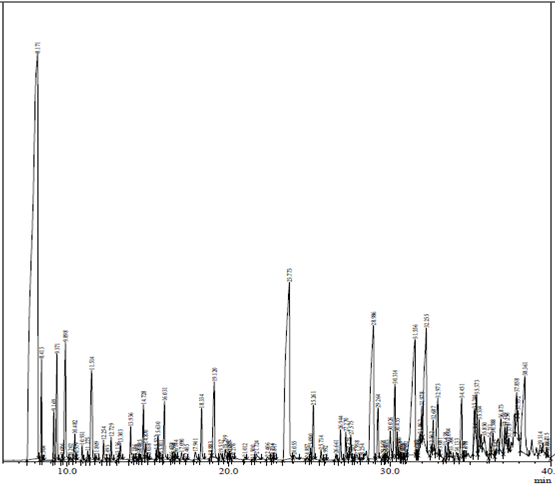


**Fig 3.3.3: Gas chromatogram of methanolic extract of *Citrus jambhiri* peels (Nainital)**

**Table 3.3.3: Chemical composition of *Citrus jambhiri*** collected from Nainital

| **S.No** | **Name** | **Area%** | **R.Time** |
| --- | --- | --- | --- |
| 1 | 2-undecanone | 0.1 | 11.718 |
| 2 | 2-Methoxy-4-vinylphenol | 0.37 | 12.025 |
| 3 | Benzene,1,4-dimethoxy-2-methyl-5-isopropyl- | 0.55 | 13.38 |
| 4 | Dodecanoic acid | 1.41 | 15.183 |
| 5 | 1,2-benzenedicarboxylic acid diethyl ester | 0.27 | 15.581 |
| 6 | 1,2,4-trimethoxy-5-[(1e)-1-propenyl]benzene | 1.79 | 15.83 |
| 7 | 11-Eicosenoic ACID (E)-,TMS (E)-,TMS derivative | 0.22 | 16.273 |
| 8 | 9-(z)-Octadecanoic acid | 1.02 | 17.482 |
| 9 | Lanceol | 0.2 | 17.629 |
| 10 | 4-Chloro-3-n-butyltetrahydropyran | 0.37 | 18.283 |
| 11 | Cyclohexanone, 3-methyl-,(2,4-dinitrophenyl)hydrazones | 0.35 | 18.395 |
| 12 | 1,8-nonadiyne | 0.17 | 18.455 |
| 13 | 1,1-Dichloro-2-propylcyclopropane | 0.33 | 19.238 |
| 14 | 2-Chloromethyl-1,3-dichloro-2-methylpropane | 0.19 | 19.433 |
| 15 | 1,2-benzenedicarboxylic acid | 15.55 | 19.585 |
| 16 | 1,1-Dichloro-2-propylcyclopropane | 1.23 | 19.828 |
| 17 | 1,1-Dichloro-2-propylcyclopropane | 0.17 | 20.228 |
| 18 | Palmitic Acid | 10.26 | 20.393 |
| 19 | 5-Chloro-2-nitrobenzyl alcohol | 0.2 | 20.575 |
| 20 | 1,1-Dichloro-2-propylcyclopropane | 0.15 | 20.667 |
| 21 | 1,1-Dichloro-2-propylcyclopropane | 0.3 | 21.148 |
| 22 | Linoelaidic acid | 10.5 | 21.221 |
| 23 | 9-(z)-Eicosenoic acid | 0.82 | 21.282 |
| 24 | 1,1-Dichloro-2-propylcyclopropane | 0.34 | 21.392 |
| 25 | Octadecanoic acid | 1.39 | 21.493 |
| 26 | 1,2-benzenedicarboxylic acid, | 1.01 | 21.891 |
| 27 | 1,3,5-trichloropent-2-ene | 0.45 | 21.99 |
| 28 | 1,1-Dichloro-2-propylcyclopropane | 0.18 | 22.215 |
| 29 | 1,1-Dichloro-2-propylcyclopropane | 0.46 | 22.317 |
| 30 | Difluorotetrachloropentanes | 0.09 | 22.767 |
| 31 | 3,5,5-Trichloro-4-methyl-2-pentanone | 0.31 | 22.965 |
| 32 | Butane, 1,2,3,4-tetrachloro- | 0.21 | 24.116 |
| 33 | Rutin | 10.33 | 25.112 |
| 34 | 4',5,6,7,8-pentamethoxyflavone | 0.46 | 33.182 |
|  | **Total** | **61.75%** |  |

**Fig 3.3.4: Gas chromatogram of methanolic extract of *Citrus jambhiri* peels (Rudraprayag)**

**Table 3.3.4: Chemical composition of *Citrus jambhiri*** collected from Rudraprayag

| **S.No** | **Name** | **Area%** | **R.Time** |
| --- | --- | --- | --- |
| 1 | 2,2-dimethylbutane | 0.48 | 4.208 |
| 2 | 2-furanmethanol | 0.15 | 4.642 |
| 3 | Cyclopent-4-ene-1,3-dione | 0.28 | 5.115 |
| 4 | 2-Hydroxy-γ-butyrolactone | 2.41 | 6.945 |
| 5 | Acetic acid | 1.37 | 8.343 |
| 6 | Cyclopropylmethanol | 0.95 | 8.712 |
| 7 | 2-acetyl-2-hydroxy-.γ.-butyrolacton | 0.47 | 9.292 |
| 8 | 2,3-dihydro-benzofuran | 0.86 | 10.612 |
| 9 | 5-hydroxymethylfurfural | 1.14 | 10.717 |
| 10 | Butane, | 1.5 | 11.04 |
| 11 | 2-Methoxy-4-vinylphenol | 4.36 | 12.024 |
| 12 | Benzaldehyde, | 0.96 | 12.635 |
| 13 | 3-Methoxybenzoic acid, 2-pentyl | 0.01 | 13.408 |
| 14 | Benzaldehyde, | 0.29 | 13.683 |
| 15 | Cytidine | 2.06 | 13.833 |
| 16 | 1-dodecanol | 0.13 | 14.15 |
| 17 | (+) Limonene | 6.30 | 14.314 |
| 18 | ß-D-glucopyranose, | 0.46 | 14.408 |
| 19 | 1,3-cyclohexadiene, | 0.46 | 14.489 |
| 20 | Cyclohexene, | 1.03 | 14.865 |
| 21 | Dodecanoic acid | 0.73 | 15.1 |
| 22 | Dodecanoic acid | 2.17 | 15.183 |
| 23 | 1,6,10-dodecatrien-3-ol, | 0.37 | 15.264 |
| 24 | Cyclooctasiloxane, | 1.44 | 16.021 |
| 25 | 2-butanone, | 0.77 | 16.244 |
| 26 | Tetradecanoic acid | 1.16 | 17.472 |
| 27 | Cyclohexasiloxane, | 1.66 | 17.724 |
| 28 | 1,2-benzenedicarboxylic | 4.52 | 18.603 |
| 29 | N-Hexadecanoic acid | 9.72 | 19.578 |
| 30 | Palmitic acid, | 0.54 | 20.394 |
| 31 | 9,12-octadecadienoic | 0.22 | 20.881 |
| 32 | 9-octadecenoic acid, | 0.33 | 20.942 |
| 33 | Linoelaidic acid, | 5.24 | 21.229 |
| 34 | 7-tetradecenal, | 4.48 | 21.283 |
| 35 | Octadecanoic acid, | 2.23 | 21.487 |
| 36 | Phenol | 0.54 | 21.688 |
| 37 | Hexadecanoic acid, | 3.65 | 25.096 |
| 38 | Di-n-Octyl phthalate | 0.58 | 25.31 |
| 39 | 1H-purin-6-amine,[(2-fluorophenyl ) | 1.43 | 25.94 |
| 40 | Hexadecanoic acid, | 2.19 | 26.985 |
| 41 | 1H-purin-6-amine, | 1.07 | 27.114 |
| 42 | 4',5,6,7,8-pentamethoxyflavone | 6.22 | 33.197 |
| 43 | 3-Fluorocholest-5-ene | 2.57 | 36.828 |
| 44 | 3',4',5,6,7,8-hexamethoxyflavone | 6.69 | 37.516 |
|  | **Total** | **81.97 %** |  |

**Fig 3.3.5: Gas chromatogram of methanolic extract of *Citrus lemon* peels (Pithoragarh)**

**Table 3.3.5: Chemical composition of *Citrus lemon*** collected from Pithoragarh

| **S.No** | **Name** | **Area%** | **R.Time** |
| --- | --- | --- | --- |
| 1 | 2,2-dimethylbutane | 0.57 | 4.219 |
| 2 | 2-furanmethanol | 0.21 | 4.654 |
| 3 | Dihydroxyacetone | 3.78 | 5.342 |
| 4 | 2,4-Dihydroxy-2,5-dimethyl-3(2H)-furan-3-one | 0.44 | 6.821 |
| 5 | 2-Hydroxy-γ-butyrolactone | 2.31 | 6.937 |
| 6 | Cyclohexene | 0.56 | 7.701 |
| 7 | Acetic acid, pentyl ester | 0.34 | 7.923 |
| 8 | Cyclopropylmethanol | 0.97 | 8.712 |
| 9 | Benzoic acid | 0.39 | 9.71 |
| 10 | (S)-(+)-2',3'-dideoxyribonolactone | 0.25 | 10.142 |
| 11 | 1,2-benzenediol | 0.5 | 10.253 |
| 12 | 2,3-dihydro-benzofuran | 0.94 | 10.611 |
| 13 | Hydroperoxide, | 1.74 | 10.751 |
| 14 | 1,2,3-propanetriol | 1.28 | 10.965 |
| 15 | 2-Methoxy-4-vinylphenol | 5.29 | 12.024 |
| 16 | Benzaldehyde, | 1.05 | 12.632 |
| 17 | Phenol, | 0.17 | 12.783 |
| 18 | Neryl | 0.87 | 12.872 |
| 19 | 1-Methyl-1-(3-methylbutyl) oxy-1-silacyclobutane | 0.91 | 13.198 |
| 20 | Guanosine | 8.86 | 13.815 |
| 21 | 1,6-octadiene, | 0.7 | 14.304 |
| 22 | β.-d-glucopyranose, | 0.64 | 14.407 |
| 23 | Dodecanoic | 2.11 | 15.177 |
| 24 | 3',5'-dimethoxyacetophenone | 0.21 | 15.252 |
| 25 | Asarone | 0.91 | 15.83 |
| 26 | Dodecanoic acid | 0.17 | 16.274 |
| 27 | Tetradecanoic acid | 1.12 | 17.47 |
| 28 | Lanceol | 0.53 | 17.626 |
| 29 | 1,6-Anhydro-.α.-d-galactofuranose | 0.88 | 17.779 |
| 30 | Mexiletine | 0.37 | 19.306 |
| 31 | N-Hexadecanoic acid | 8.46 | 19.575 |
| 32 | Palmitic Acid | 10.3 | 20.397 |
| 33 | 11,14-Eicosadienoic acid | 0.14 | 20.883 |
| 34 | Linoelaidic acid | 4.22 | 21.227 |
| 35 | 7-(z)-tetradecenal | 3.51 | 21.281 |
| 36 | Rutin | 2.08 | 21.485 |
| 37 | Dodecanamide, N-(2-hydroxyethyl) | 0.16 | 23.278 |
| 38 | Squalene | 0.15 | 27.978 |
| 39 | Hesperetin | 4.68 | 30.9 |
| 40 | D L-α-tocopherol | 0.28 | 33.34 |
| 41 | 4',5,6,7,8-pentamethoxyflavone | 6.38 | 35.486 |
|  | **Total** | **79.49%** |  |

**Fig 3.3.6: Gas chromatogram of methanolic extract of *Citrus lemon* peels (Almora)**

**Table 3.3.6: Chemical composition of *Citrus lemon*** collected from Almora

| **S.No** | **Name** | **Area%** | **R.Time** |
| --- | --- | --- | --- |
| 1 | 2,2-dimethylbutane | 0.57 | 4.218 |
| 2 | 2-Furanmethanol | 0.21 | 4.65 |
| 3 | Dihydroxyacetone | 3.67 | 5.312 |
| 4 | 1,2,3-Propanetriol,1-acetate | 0.38 | 6.229 |
| 5 | 2,4-Dihydroxy-2,5-dimethyl-3(2H)-furan-3-one | 0.37 | 6.821 |
| 6 | 2-Hydroxy-γ-butyrolactone | 2.37 | 6.933 |
| 7 | Acetic acid, pentyl ester | 0.31 | 7.922 |
| 8 | 2,5-Anhydro-1,6-dideoxyhexo-3,4-diulose | 0.26 | 8.008 |
| 9 | Acetophenone | 2.85 | 8.289 |
| 10 | Cyclopropylmethanol | 0.89 | 8.712 |
| 11 | 1-[(Thiomorpholin-4-yl) carboximidoyl] guanidine | 0.17 | 9.167 |
| 12 | 2-acetyl-2-hydroxy γ butyrolacton | 0.54 | 9.289 |
| 13 | (+) Limonene | 1.15 | 9.709 |
| 14 | 2-pentanone | 0.87 | 10.36 |
| 15 | 2,3-dihydro-benzofuran | 0.82 | 10.612 |
| 16 | Hydroperoxide,1-methylpentyl | 1.5 | 10.745 |
| 17 | 2-Methoxy-4-vinylphenol | 4.58 | 12.024 |
| 18 | Anisaldehyde Dimethyl acetal | 0.28 | 12.769 |
| 19 | Guanosine | 9.93 | 13.815 |
| 20 | Pentafluoropropionic acid | 0.23 | 14.161 |
| 21 | 1,5-octadien-3,7-diol, 3,7-dimethyl- (terpenoides) | 0.71 | 14.304 |
| 22 | D-Allose | 0.61 | 14.395 |
| 23 | Dodecanoic acid | 2 | 15.175 |
| 24 | Tetradecanoic acid | 1.06 | 17.467 |
| 25 | Cis –Lanceol | 0.53 | 17.625 |
| 26 | n-Hexadecanoic acid | 6.94 | 19.572 |
| 27 | 4,7-Di(methyloxy) coumarin | 0.27 | 19.893 |
| 28 | Palmitic Acid | 0.24 | 20.395 |
| 29 | 8,11,14-Eicosatrienoic acid | 0.1 | 20.942 |
| 30 | Linoelaidic acid | 4.06 | 21.225 |
| 31 | 7-Tetradecenal | 3 | 21.277 |
| 32 | Octadecanoic acid | 1.12 | 21.483 |
| 33 | Decanal | 0.2 | 25.311 |
| 34 | Hexadecanoic acid, 2-hydroxy-(hydroxymethyl)ethyl ester | 1.31 | 26.987 |
| 35 | Squalene | 0.13 | 27.979 |
| 36 | 4',5,6,7,8-Pentamethoxyflavone | 7.28 | 33.2 |
| 37 | Rutin | 2.72 | 36.828 |
| 38 | 3',4',5,6,7,8-Hexamethoxyflavone | 8.43 | 37.518 |
|  | **Total** | **71.85 %** |  |

**Fig 3.3.7: Gas chromatogram Fig 4.7 Gas chromatogram of methanolic extract of *Citrus lemon* peels (Nainital)**

**Table 3.3.7: Chemical composition of *Citrus lemon*** collected from Nainital

| **S.No** | **Name** | **Area%** | **R.Time** |
| --- | --- | --- | --- |
| 1 | Acetic acid, pentyl ester | 0.34 | 7.923 |
| 2 | Cyclopropylmethanol | 0.97 | 8.712 |
| 3 | Benzoic acid | 0.39 | 9.71 |
| 4 | (S)-(+)-2',3'-dideoxyribonolactone | 0.25 | 10.142 |
| 5 | 1,2-benzenediol | 0.5 | 10.253 |
| 6 | 2,3-dihydro-benzofuran | 0.94 | 10.611 |
| 7 | Hydroperoxide, | 1.74 | 10.751 |
| 8 | 1,2,3-propanetriol | 1.28 | 10.965 |
| 9 | 2-Methoxy-4-vinylphenol | 5.29 | 12.024 |
| 10 | Benzaldehyde, | 1.05 | 12.632 |
| 11 | Phenol, | 0.17 | 12.783 |
| 12 | Neryl | 0.87 | 12.872 |
| 13 | 1-Methyl-1-(3-methylbutyl) oxy-1-silacyclobutane | 0.91 | 13.198 |
| 14 | Guanosine | 8.86 | 13.815 |
| 15 | 1,6-octadiene, | 0.7 | 14.304 |
| 16 | .Beta.-d-glucopyranose, | 0.64 | 14.407 |
| 17 | Dodecanoic | 2.11 | 15.177 |
| 18 | 3',5'-dimethoxyacetophenone | 0.21 | 15.252 |
| 19 | Asarone | 0.91 | 15.83 |
| 20 | Dodecanoic acid | 0.17 | 16.274 |
| 21 | Tetradecanoic acid | 1.12 | 17.47 |
| 22 | Lanceol | 0.53 | 17.626 |
| 23 | 1,6-Anhydro-.α.-d-galactofuranose | 0.88 | 17.779 |
| 24 | Mexiletine | 0.37 | 19.306 |
| 25 | N-Hexadecanoic acid | 8.46 | 19.575 |
| 26 | Palmitic Acid | 0.3 | 20.397 |
| 27 | 11,14-Eicosadienoic acid | 0.14 | 20.883 |
| 28 | Linoelaidic acid | 4.22 | 21.227 |
| 29 | 7-(z)-tetradecenal | 3.51 | 21.281 |
| 30 | (+) Limonene | 2.24 | 21.485 |
| 31 | Dodecanamide, N-(2-hydroxyethyl) | 0.16 | 23.278 |
| 32 | Squalene | 0.15 | 27.978 |
| 33 | Hesperetin | 0.68 | 28.34 |
| 34 | D L-α-tocopherol | 0.28 | 30.120 |
| 35 | 4',5,6,7,8-pentamethoxyflavone | 6.38 | 31.123 |
| 36 | 2,2-dimethylbutane | 0.57 | 34.219 |
| 37 | 2-furanmethanol | 0.21 | 34.654 |
| 38 | Dihydroxyacetone | 3.78 | 35.342 |
| 39 | 2,4-Dihydroxy-2,5-dimethyl-3(2H)-furan-3-one | 0.44 | 36.821 |
| 40 | 2-Hydroxy-γ-butyrolactone | 2.31 | 36.937 |
| 41 | Cyclohexene | 0.56 | 37.701 |
|  | **Total** | **65.43 %** |  |

**Fig 3.3.8: Gas chromatogram of methanolic extract of *Citrus lemon* peels (Rudraprayag)**

**Table 3.3.8: Chemical composition of *Citrus lemon*** collected from Rudraprayag

| **S.No** | **Name** | **Area%** | **R.Time** |
| --- | --- | --- | --- |
| 1 | 2,2-dimethylbutane | 0.39 | 4.22 |
| 2 | Cycloserine | 0.14 | 5.037 |
| 3 | Dihydroxyacetone | 2.97 | 5.33 |
| 4 | 2-hydroxy-2-Cyclopenten-1-one | 1.98 | 5.817 |
| 5 | Acetic acid | 0.3 | 6.23 |
| 6 | 2,4-Dihydroxy-2,5-dimethyl-3(2H)-furan-3-one | 0.32 | 6.821 |
| 7 | 2-Hydroxy-γ-butyrolactone | 2.12 | 6.936 |
| 8 | Cyclohexene | 0.94 | 7.699 |
| 9 | Methyl 2-oxopropanoate | 0.25 | 7.92 |
| 10 | Cyclopentane | 1.37 | 8.338 |
| 11 | Cyclopropylmethanol | 0.86 | 8.711 |
| 12 | 2-acetyl-2-hydroxy-γ-butyrolacton | 0.47 | 9.288 |
| 13 | Rutin | 1.29 | 9.519 |
| 14 | Benzoic acid | 0.37 | 9.72 |
| 15 | (S)-(+)-2',3'-Dideoxyribonolactone | 0.2 | 10.142 |
| 16 | 2,3-dihydro-benzofuran | 0.9 | 10.611 |
| 17 | 1,2,3-propanetriol | 0.77 | 10.962 |
| 18 | Acetic acid | 0.23 | 11.573 |
| 19 | 2-Methoxy-4-vinylphenol | 4.56 | 12.024 |
| 20 | Guanosine | 10.41 | 13.816 |
| 21 | (+) Limonene | 3.45 | 14.313 |
| 22 | Beta-d-glucopyranose | 0.94 | 14.42 |
| 23 | Dodecanoic acid | 11.56 | 15.208 |
| 24 | 1,2- diethyl benzenedicarboxylic acid | 0.32 | 15.577 |
| 25 | 3-Deoxy-d-mannoic lactone | 0.81 | 15.752 |
| 26 | Tetradecanoic acid | 3.9 | 17.478 |
| 27 | Cis-Lanceol | 0.36 | 17.625 |
| 28 | 1,6-Anhydro-α-d-galactofuranose | 1.15 | 17.773 |
| 29 | 1-Methyl-4-phenyl-4-propionoxypiperidine | 0.26 | 18.253 |
| 30 | Myristic acid | 0.17 | 18.426 |
| 31 | 2,2-Bis(n-propyl) malonodinitrile | 0.38 | 18.873 |
| 32 | Mexiletine | 0.69 | 19.298 |
| 33 | n-Hexadecanoic acid | 7.27 | 19.573 |
| 34 | Palmitic Acid | 0.15 | 20.394 |
| 35 | Z,Z-8,10-Hexadecadien-1-ol | 0.15 | 20.883 |
| 36 | Linoelaidic acid | 3.48 | 21.224 |
| 37 | 9-(Z)-Eicosenoic acid | 3.5 | 21.278 |
| 38 | Octadecanoic acid | 0.97 | 21.483 |
| 39 | Decanal | 0.17 | 25.311 |
| 40 | Hesperetin | 0.67 | 30.322 |
| 41 | 4',5,6,7,8-Pentamethoxyflavone | 6.89 | 33.198 |
| 42 | 3',4',5,6,7,8-Hexamethoxyflavone | 8.3 | 37.528 |
|  | **Total** | **83.37 %** |  |

**Fig 3.3.9: Gas chromatogram of methanolic extract of *Citrus sinensis* peels (Pithoragarh)**

**Table 3.3.9: Chemical composition of *Citrus sinensis*** collected from Pithoragarh

| **S.No** | **Name** | **Area%** | **R.Time** |
| --- | --- | --- | --- |
| 1 | 2-furanmethanol | 0.86 | 4.673 |
| 2 | 1,3-cyclopentenedione | 1.33 | 5.118 |
| 3 | Α-Beta-crotonolactone | 0.63 | 5.583 |
| 4 | (+)-limonene | 2.05 | 7.695 |
| 5 | Acetophenone | 1.07 | 8.29 |
| 6 | 2-Hexanone | 1.76 | 8.449 |
| 7 | Pentanal | 1.21 | 8.76 |
| 8 | 2-acetyl-2-hydroxy-.γ.-butyrolacton | 0.77 | 9.383 |
| 9 | 1,5-anhydro-6-deoxyhexo-2,3-diulose | 3.46 | 9.596 |
| 10 | Benzoic acid | 0.62 | 9.808 |
| 11 | 2,3-dihydro-benzofuran | 0.57 | 10.619 |
| 12 | 5-hydroxymethylfurfural | 1.99 | 10.778 |
| 13 | 1,2,3-propanetriol | 2.64 | 11.113 |
| 14 | 2-Methoxy-4-vinylphenol | 4.39 | 12.033 |
| 15 | Benzaldehyde | 1.47 | 12.672 |
| 16 | Dodecanoic acid | 1.24 | 15.201 |
| 17 | Tetradecanoic acid | 0.96 | 17.482 |
| 18 | 1,6-Anhydro-α-d-galactofuranose | 1.55 | 17.895 |
| 19 | Hexadecanoic acid | 0.59 | 19.239 |
| 20 | N-Hexadecanoic acid acid | 8.27 | 19.602 |
| 21 | 9,12-Octadecadienoic acid | 0.43 | 20.881 |
| 22 | Linoelaidic acid | 10.22 | 21.259 |
| 23 | 9-octadecenoic acid | 0.99 | 21.497 |
| 24 | 8-hydroxysclerodin methyl ether | 0.86 | 32.797 |
| 25 | 4',5,6,7,8-pentamethoxyflavone | 9.17 | 33.286 |
| 26 | Rutin | 5.46 | 36.909 |
| 27 | 3',4',5,6,7,8-hexamethoxyflavone | 12.45 | 37.7 |
|  | **Total** | **77.01 %** |  |

**Fig 3.3.10: Gas chromatogram of methanolic extract of *Citrus sinensis* peels (Almora)**

**Table 3.3.10: Chemical composition of *Citrus sinensis*** collected from Almora

| **S.No** | **Name** | **Area%** | **R.Time** |
| --- | --- | --- | --- |
| 1 | 1,3-cyclopentenedione | 0.21 | 5.11 |
| 2 | Dihydroxyacetone | 1.64 | 5.321 |
| 3 | Phenol | 0.89 | 6.758 |
| 4 | 2-Hydroxy-γ-butyrolactone | 1.64 | 6.938 |
| 5 | Octanal | 1 | 7.202 |
| 6 | Pentanal | 0.85 | 8.705 |
| 7 | 1-Butyl(dimethyl) silyloxypropane | 0.25 | 9.175 |
| 8 | 2-acetyl-2-hydroxy γ butyrolactone | 0.18 | 9.289 |
| 9 | Azulene | 1.16 | 10.261 |
| 10 | Decanal | 2.64 | 10.463 |
| 11 | 2,3-dihydro-benzofuran | 0.8 | 10.611 |
| 12 | (+) Limonene | 3.04 | 11.718 |
| 13 | 2-Furanacetic acid | 0.2 | 11.95 |
| 14 | 2-Methoxy-4-vinylphenol | 3.77 | 12.024 |
| 15 | Resorcinol | 0.26 | 12.425 |
| 16 | 4-hydroxybenzaldehyde | 1.03 | 12.861 |
| 17 | 2-phenylisopropanol | 0.24 | 13.13 |
| 18 | 1-chloro-4-methoxy Benzene | 0.24 | 13.192 |
| 19 | Sucrose | 10.39 | 13.65 |
| 20 | Decanoic acid | 0.12 | 13.903 |
| 21 | 1-chloro-Hexadecane | 0.27 | 14.15 |
| 22 | 1-chloro-4-methoxy-Benzene | 0.65 | 14.313 |
| 23 | 2-Ketoisocaproic acid | 0.67 | 14.403 |
| 24 | Farnesol | 0.7 | 14.503 |
| 25 | 2-Oxabicyclo[2.2.2]octan-6-ol, 1,3,3-trimethyl acetate | 1.45 | 14.606 |
| 26 | Aceburic acid | 0.17 | 15.02 |
| 27 | 2-Ketoisocaproic acid | 0.51 | 15.116 |
| 28 | Decanoic acid | 1 | 15.186 |
| 29 | Octadecanoic acid | 0.64 | 15.268 |
| 30 | 1,2-Benzenedicarboxylic acid, | 0.13 | 15.5 |
| 31 | Hexanoic acid | 0.24 | 15.757 |
| 32 | 2,E-Prostaglandin | 0.31 | 16.508 |
| 33 | 2-Hydroxyisocaproic acid, trimethylsilyl ester | 0.3 | 17.118 |
| 34 | Tetradecanoic acid | 0.57 | 17.477 |
| 35 | 2,3-dimethyl-4-quinolinone | 0.15 | 18.242 |
| 36 | Myristic acid | 0.88 | 18.429 |
| 37 | Eicosanoic acid | 1.02 | 19.242 |
| 38 | N-Hexadecanoic acid | 5.54 | 19.578 |
| 39 | Hexadecanoic acid, trimethylsilyl ester | 4.69 | 20.395 |
| 40 | Petasitene | 0.2 | 21.067 |
| 41 | Eicosanoic acid, methyl ester | 0.06 | 21.175 |
| 42 | Linoelaidic acid | 1.84 | 21.23 |
| 43 | 7-(z)-tetradecenal | 2.03 | 21.284 |
| 44 | Octadecanoic acid | 0.7 | 21.489 |
| 45 | Linoelaidic acid | 8.26 | 21.921 |
| 46 | Rutin | 5.68 | 24.99 |
|  | **Total** | **66.37 %** |  |

**Fig 3.3.11: Gas chromatogram of methanolic extract of *Citrus sinensis* peels (Nainital)**

**Table 3.3.11: Chemical composition of *Citrus sinensis*** collected from Nainital

| **S.No** | **Name** | **Area%** | **R.Time** |
| --- | --- | --- | --- |
| 1 | Dihydroxyacetone | 2.97 | 5.33 |
| 2 | 2-hydroxy-2-Cyclopenten-1-one | 1.98 | 5.817 |
| 3 | Acetic acid | 0.3 | 6.23 |
| 4 | 2,4-Dihydroxy-2,5-dimethyl-3(2H)-furan-3-one | 0.32 | 6.821 |
| 5 | 2-Hydroxy-γ-butyrolactone | 2.12 | 6.936 |
| 6 | Cyclohexene | 0.94 | 7.699 |
| 7 | Methyl 2-oxopropanoate | 0.25 | 7.92 |
| 8 | Cyclopentane | 1.37 | 8.338 |
| 9 | Cyclopropylmethanol | 0.86 | 8.711 |
| 10 | 2-acetyl-2-hydroxy-γ-butyrolacton | 0.47 | 9.288 |
| 11 | Rutin | 1.29 | 9.519 |
| 12 | Benzoic acid | 0.37 | 9.72 |
| 13 | (S)-(+)-2',3'-Dideoxyribonolactone | 0.2 | 10.142 |
| 14 | 2,3-dihydro-benzofuran | 0.9 | 10.611 |
| 15 | 1,2,3-propanetriol | 0.77 | 10.962 |
| 16 | (+) Limonene | 1.04 | 11.573 |
| 17 | 2-Methoxy-4-vinylphenol | 4.56 | 12.024 |
| 18 | Guanosine | 10.41 | 13.816 |
| 19 | 1,6-Octadiene | 0.44 | 14.313 |
| 20 | Beta-d-glucopyranose | 0.94 | 14.42 |
| 21 | Dodecanoic acid | 11.56 | 15.208 |
| 22 | 1,2- diethyl benzenedicarboxylic acid | 0.32 | 15.577 |
| 23 | 3-Deoxy-d-mannoic lactone | 0.81 | 15.752 |
| 24 | Tetradecanoic acid | 3.9 | 17.478 |
| 25 | Cis-Lanceol | 0.36 | 17.625 |
| 26 | 1,6-Anhydro-α-d-galactofuranose | 1.15 | 17.773 |
| 27 | 1-Methyl-4-phenyl-4-propionoxypiperidine | 0.26 | 18.253 |
| 28 | Myristic acid | 10.17 | 18.426 |
| 29 | 2,2-Bis(n-propyl) malonodinitrile | 0.38 | 18.873 |
| 30 | Mexiletine | 0.69 | 19.298 |
| 31 | n-Hexadecanoic acid | 7.27 | 19.573 |
| 32 | Palmitic Acid | 0.15 | 20.394 |
| 33 | Z,Z-8,10-Hexadecadien-1-ol | 0.15 | 20.883 |
| 34 | Linoelaidic acid | 3.48 | 21.224 |
| 35 | 9-(Z)-Eicosenoic acid | 3.5 | 21.278 |
| 36 | Octadecanoic acid | 0.97 | 21.483 |
| 37 | Decanal | 0.17 | 25.311 |
| 38 | Hesperetin | 0.67 | 30.322 |
| 39 | 4',5,6,7,8-Pentamethoxyflavone | 6.89 | 33.198 |
|  | **Total** | **84.54%** |  |

**Fig 3.3.12: Gas chromatogram of Fig: 4.12 Gas chromatogram of methanolic extract of *Citrus sinensis* peels (Rudraprayag)**

**Table 3.3.12: Chemical composition of *Citrus sinensis*** collected from Rudraprayag

| **S.No** | **Name** | **Area%** | **R.Time** |
| --- | --- | --- | --- |
| 1 | 2,2- Dichlorobutane | 0.38 | 4.28 |
| 2 | Acetonyl acetate | 0.27 | 4.839 |
| 3 | Cyclopent-4-ene-1,3-dione | 0.32 | 5.117 |
| 4 | Dihydroxyacetone | 2.95 | 5.295 |
| 5 | 2-shydroxy-γ-butyrolactone | 3.01 | 6.931 |
| 6 | (+)-Limonene | 0.61 | 7.691 |
| 7 | Cyclopropylmethanol | 1.26 | 8.708 |
| 8 | Octanoic acid | 0.39 | 9.832 |
| 9 | Α-ketoisovaleric acid | 0.28 | 10.433 |
| 10 | Geranial | 0.83 | 10.612 |
| 11 | 2-undecanone | 1.84 | 11.719 |
| 12 | 2-methoxy-4-vinylphenol | 4.29 | 12.024 |
| 13 | Decanoic acid | 1.11 | 12.642 |
| 14 | Linalool | 0.6 | 13.195 |
| 15 | Cytidine | 4.04 | 13.815 |
| 16 | 2-tridecanone | 2.32 | 14.421 |
| 17 | 2-hydroxyisocaproic acid | 0.95 | 14.506 |
| 18 | Undecanoic acid | 0.31 | 15.108 |
| 19 | Dodecanoic acid | 17.31 | 15.225 |
| 20 | L-(+)-lactic acid | 7.46 | 15.756 |
| 21 | Hexanoic acid, tms derivative | 1.71 | 15.951 |
| 22 | Dodecanoic acid | 0.31 | 16.278 |
| 23 | Tetradecanoic acid | 5.4 | 17.485 |
| 24 | Myristic acid, | 3.35 | 18.429 |
| 25 | 5-(2,4-dimethoxyphenyl)cyclohexane-1,3-dione | 0.51 | 18.872 |
| 26 | Octadecanoic acid, methyl ester | 0.8 | 19.243 |
| 27 | N-hexadecanoic acid | 7.71 | 19.578 |
| 28 | Palmitic acid | 3.6 | 20.395 |
| 29 | Linoelaidic acid | 2.13 | 21.226 |
| 30 | Cis-11-Eicosenoic acid | 3.64 | 21.282 |
| 31 | Octadecanoic acid | 0.8 | 21.488 |
| 32 | 9,12-(z,z)- octadecadienoic acid | 0.22 | 21.922 |
| 33 | 9-octadecenoic acid | 0.38 | 21.973 |
| 34 | Stearic acid | 0.45 | 22.2 |
| 35 | 4',5,6,7,8-pentamethoxyflavone | 3.75 | 33.187 |
| 36 | Γ-sitosterol | 2.21 | 36.83 |
| 37 | 3',4',5,6,7,8-hexamethoxyflavone | 3.43 | 37.496 |
| 38 | Monoterpenes | 3.46 | 44.21 |
| 39 | Rutin | 3.06 | 46.21 |
|  | **Total** | **97.45 %** |  |

**Fig 3.3.13: Gas chromatogram of methanolic extract of *Citrus aurentifolia* peels (Pithoragarh)**

**Table 3.3.13: Chemical composition of *Citrus aurentifolia* collected from Pithoragarh**

| **S.No** | **Name** | **Area%** | **R.Time** |
| --- | --- | --- | --- |
| 1 | Acetic acid, pentyl ester | 0.38 | 4.28 |
| 2 | Cyclopropylmethanol | 0.27 | 4.839 |
| 3 | Benzoic acid | 0.32 | 5.117 |
| 4 | (S)-(+)-2',3'-dideoxyribonolactone | 2.95 | 5.295 |
| 5 | 1,2-benzenediol | 3.01 | 6.931 |
| 6 | 2,3-dihydro-benzofuran | 0.61 | 7.691 |
| 7 | Hydroperoxide, | 1.26 | 8.708 |
| 8 | 1,2,3-propanetriol | 0.39 | 9.832 |
| 9 | 2-Methoxy-4-vinylphenol | 0.28 | 10.433 |
| 10 | (+) Limonene | 1.45 | 10.612 |
| 11 | Phenol, | 1.84 | 11.719 |
| 12 | Neryl | 4.29 | 12.024 |
| 13 | 1-Methyl-1-(3-methylbutyl)oxy-1-silacyclobutane | 1.11 | 12.642 |
| 14 | Guanosine | 0.6 | 13.195 |
| 15 | 1,6-octadiene, | 4.04 | 13.815 |
| 16 | .Beta.-d-glucopyranose, | 2.32 | 14.421 |
| 17 | Dodecanoic | 0.95 | 14.506 |
| 18 | 3',5'-dimethoxyacetophenone | 0.31 | 15.108 |
| 19 | Asarone | 11.31 | 15.225 |
| 20 | Dodecanoic acid | 0.46 | 15.756 |
| 21 | Tetradecanoic acid | 1.71 | 15.951 |
| 22 | Lanceol | 10.31 | 16.278 |
| 23 | 1,6-Anhydro-.α.-d-galactofuranose | 5.4 | 17.485 |
| 24 | Mexiletine | 3.35 | 18.429 |
| 25 | N-Hexadecanoic acid | 0.51 | 18.872 |
| 26 | Palmitic Acid | 0.8 | 19.243 |
| 27 | 11,14-Eicosadienoic acid | 7.71 | 19.578 |
| 28 | Linoelaidic acid | 3.6 | 20.395 |
| 29 | 7-(z)-tetradecenal | 2.13 | 21.226 |
| 30 | Octadecanoic acid | 3.64 | 21.282 |
| 31 | Dodecanamide, N-(2-hydroxyethyl) | 0.8 | 21.488 |
| 32 | Squalene | 0.22 | 21.922 |
| 33 | Hesperetin | 0.38 | 21.973 |
| 34 | D L-α-tocopherol | 0.45 | 22.2 |
| 35 | 4',5,6,7,8-pentamethoxyflavone | 0.75 | 33.187 |
| 36 | 2,2-dimethylbutane | 2.21 | 36.83 |
| 37 | 2-furanmethanol | 3.43 | 37.496 |
| 38 | Dihydroxyacetone | 0.46 | 44.21 |
| 39 | 2,4-Dihydroxy-2,5-dimethyl-3(2H)-furan-3-one | 3.23 | 46.89 |
| 40 | 2-Hydroxy-γ-butyrolactone | 3.12 | 47.78 |
| 41 | Cyclohexene | 0.67 | 50.54 |
|  | **Total** | **92.41 %** |  |

**Fig 3.3.14: Gas chromatogram of methanolic extract of *Citrus aurentifolia* peels (Almora)**

**Table 3.3.14: Chemical composition of *Citrus aurentifolia*** collected from Almora

| **S.No** | **Name** | **Area%** | **R.Time** |
| --- | --- | --- | --- |
| 1 | Dihydroxyacetone | 0.18 | 0.31 |
| 2 | 2-hydroxy-2-Cyclopenten-1-one | 0.25 | 0.38 |
| 3 | Acetic acid | 0.18 | 0.27 |
| 4 | 2,4-Dihydroxy-2,5-dimethyl-3(2H)-furan-3-one | 0.21 | 0.32 |
| 5 | 2-Hydroxy-γ-butyrolactone | 1.64 | 2.95 |
| 6 | Cyclohexene | 1.24 | 1.8 |
| 7 | (+) Limonene | 3.56 | 3.01 |
| 8 | Cyclopentane | 0.89 | 0.61 |
| 9 | Cyclopropylmethanol | 0.12 | 0.73 |
| 10 | 2-acetyl-2-hydroxy-γ-butyrolacton | 1.64 | 1.26 |
| 11 | Rutin | 1 | 0.4 |
| 12 | Benzoic acid | 0.57 | 0.24 |
| 13 | (S)-(+)-2',3'-Dideoxyribonolactone | 0.22 | 1.02 |
| 14 | 2,3-dihydro-benzofuran | 0.4 | 0.39 |
| 15 | 1,2,3-propanetriol | 0.27 | 0.28 |
| 16 | Acetic acid | 0.27 | 0.83 |
| 17 | 2-Methoxy-4-vinylphenol | 0.85 | 0.54 |
| 18 | Guanosine | 0.25 | 1.84 |
| 19 | 1,6-Octadiene | 0.18 | 0.37 |
| 20 | Beta-d-glucopyranose | 0.92 | 4.29 |
| 21 | Dodecanoic acid | 1.16 | 1.11 |
| 22 | 1,2- diethyl benzenedicarboxylic acid | 0.39 | 0.6 |
| 23 | 3-Deoxy-d-mannoic lactone | 2.64 | 4.04 |
| 24 | Tetradecanoic acid | 0.8 | 0.28 |
| 25 | Cis-Lanceol | 0.54 | 2.32 |
| 26 | 1,6-Anhydro-α-d-galactofuranose | 0.26 | 0.95 |
| 27 | 1-Methyl-4-phenyl-4-propionoxypiperidine | 0.27 | 0.31 |
| 28 | Myristic acid | 0.2 | 17.31 |
| 29 | 2,2-Bis(n-propyl) malonodinitrile | 0.2 | 0.46 |
| 30 | Mexiletine | 3.77 | 1.71 |
| 31 | n-Hexadecanoic acid | 0.26 | 10.31 |
| 32 | Palmitic Acid | 0.95 | 5.4 |
| 33 | Z,Z-8,10-Hexadecadien-1-ol | 0.4 | 0.4 |
| 34 | Linoelaidic acid | 1.03 | 3.35 |
| 35 | 9-(Z)-Eicosenoic acid | 0.24 | 0.51 |
| 36 | Octadecanoic acid | 0.24 | 0.8 |
| 37 | Decanal | 1.39 | 7.71 |
| 38 | Hesperetin | 0.12 | 3.6 |
| 39 | 4',5,6,7,8-Pentamethoxyflavone | 0.27 | 2.13 |
| 40 | Geranial | 8.67 | 0.8 |
| 41 | 2-undecanone | 0.7 | 0.22 |
| 42 | 2-methoxy-4-vinylphenol | 1.45 | 0.38 |
| 43 | Decanoic acid | 0.17 | 0.45 |
| 44 | Linalool | 10.51 | 3.75 |
| 45 | Cytidine | 1 | 2.21 |
| 46 | 2-tridecanone | 0.64 | 3.43 |
| 47 | L-(+)-lactic acid | 7.067 | 4.890 |
|  | **Total** | **57.287 %** |  |

**Fig 3.3.15: Gas chromatogram of methanolic extract of *Citrus aurentifolia* peels (Nainital)**

**Table 3.3.15: Chemical composition of *Citrus aurentifolia*** collected from Nainital

| **S.No** | **Name** | **Area%** | **R.Time** |
| --- | --- | --- | --- |
| 1 | 2-furanmethanol | 0.21 | 4.627 |
| 2 | Propanoic acid, 2-methyl ester | 0.54 | 5.009 |
| 3 | Cyclopent-4-ene-1,3-dione | 0.18 | 5.101 |
| 4 | Dihydroxyacetone | 2.4 | 5.331 |
| 5 | 2,4-dihydroxy-2,5-dimethyl-3(2h)-furan-3-one | 0.29 | 6.801 |
| 6 | Γ-butyrolacton | 0.66 | 6.939 |
| 7 | (+)-Limonene | 1.24 | 7.678 |
| 8 | 1-phenylethanone | 0.31 | 8.279 |
| 9 | Acetic acid, 1-(2-methyltetrazol-5-yl)ethenyl ester | 0.97 | 8.354 |
| 10 | Cyclopropylmethanol | 0.45 | 8.706 |
| 11 | 1,5-anhydro-6-deoxyhexo-2,3-diulose | 1.02 | 9.511 |
| 12 | Benzoic acid | 1.93 | 9.736 |
| 13 | 2,3-dihydro-benzofuran | 0.92 | 10.61 |
| 14 | 1,2,3-propanetriol | 1.12 | 10.992 |
| 15 | Linalyl acetate | 0.2 | 11.094 |
| 16 | 2-undecanone | 2.61 | 11.667 |
| 17 | 2-methoxy-4-vinylphenol | 3.82 | 12.023 |
| 18 | Benzaldehyde | 0.98 | 12.634 |
| 19 | Cytidine | 0.6 | 13.803 |
| 20 | Vinyldecanoate | 0.3 | 14.131 |
| 21 | 3-hydroxydecanoic acid | 0.37 | 16.148 |
| 22 | Dodecanoic ethenyl ester | 0.8 | 16.628 |
| 23 | Tetradecanoic acid | 0.42 | 17.465 |
| 24 | α-d-glucopyranose | 0.49 | 17.771 |
| 25 | Mexiletine | 0.3 | 19.311 |
| 26 | N-hexadecanoic acid | 4.52 | 19.573 |
| 27 | Phytol | 10.29 | 21.04 |
| 28 | Linoelaidic acid | 3.84 | 21.23 |
| 29 | Octadecanoic acid | 0.5 | 21.481 |
| 30 | 4',5,6,7,8-pentamethoxyflavone | 7.62 | 33.226 |
| 31 | 3',4',5,6,7,8-hexamethoxyflavone | 8.33 | 37.571 |
| 32 | Rutin | 9.62 | 40.561 |
|  | **Total** | **67.85 %** |  |

**Fig 3.3.16: Gas chromatogram of methanolic extract of *Citrus aurentifolia* peels (Rudraprayag)**

**Table 3.3.16: Chemical composition of *Citrus aurentifolia* collected from Rudraprayag**

| **S.No** | **Name** | **Area%** | **R.Time** |
| --- | --- | --- | --- |
| 1 | 2,2-dimethylbutane | 0.39 | 4.22 |
| 2 | Cycloserine | 0.14 | 5.037 |
| 3 | Dihydroxyacetone | 2.97 | 5.33 |
| 4 | 2-hydroxy-2-Cyclopenten-1-one | 1.98 | 5.817 |
| 5 | Acetic acid | 0.3 | 6.23 |
| 6 | 2,4-Dihydroxy-2,5-dimethyl-3(2H)-furan-3-one | 0.32 | 6.821 |
| 7 | 2-Hydroxy-γ-butyrolactone | 2.12 | 6.936 |
| 8 | Cyclohexene | 0.94 | 7.699 |
| 9 | Methyl 2-oxopropanoate | 0.25 | 7.92 |
| 10 | Cyclopentane | 1.37 | 8.338 |
| 11 | Cyclopropylmethanol | 0.86 | 8.711 |
| 12 | 2-acetyl-2-hydroxy-γ-butyrolacton | 0.47 | 9.288 |
| 13 | Rutin | 1.29 | 9.519 |
| 14 | Benzoic acid | 0.37 | 9.72 |
| 15 | (S)-(+)-2',3'-Dideoxyribonolactone | 0.2 | 10.142 |
| 16 | 2,3-dihydro-benzofuran | 0.9 | 10.611 |
| 17 | 1,2,3-propanetriol | 0.77 | 10.962 |
| 18 | (+) Limonene | 2.34 | 11.573 |
| 19 | 2-Methoxy-4-vinylphenol | 4.56 | 12.024 |
| 20 | Guanosine | 10.41 | 13.816 |
| 21 | 1,6-Octadiene | 0.44 | 14.313 |
| 22 | Beta-d-glucopyranose | 0.94 | 14.42 |
| 23 | Dodecanoic acid | 11.56 | 15.208 |
| 24 | 1,2- diethyl benzenedicarboxylic acid | 0.32 | 15.577 |
| 25 | 3-Deoxy-d-mannoic lactone | 0.81 | 15.752 |
| 26 | Tetradecanoic acid | 3.9 | 17.478 |
| 27 | Cis-Lanceol | 0.36 | 17.625 |
| 28 | 1,6-Anhydro-α-d-galactofuranose | 1.15 | 17.773 |
| 29 | 1-Methyl-4-phenyl-4-propionoxypiperidine | 0.26 | 18.253 |
| 30 | Myristic acid | 0.17 | 18.426 |
| 31 | 2,2-Bis(n-propyl) malonodinitrile | 0.38 | 18.873 |
| 32 | Mexiletine | 0.69 | 19.298 |
| 33 | n-Hexadecanoic acid | 7.27 | 19.573 |
| 34 | Palmitic Acid | 10.15 | 20.394 |
| 35 | Z,Z-8,10-Hexadecadien-1-ol | 0.15 | 20.883 |
| 36 | Linoelaidic acid | 3.48 | 21.224 |
| 37 | 9-(Z)-Eicosenoic acid | 3.5 | 21.278 |
| 38 | Octadecanoic acid | 0.97 | 21.483 |
| 39 | Decanal | 0.17 | 25.311 |
| 40 | Hesperetin | 0.67 | 30.322 |
| 41 | 4',5,6,7,8-Pentamethoxyflavone | 6.89 | 33.198 |
| 42 | 3',4',5,6,7,8-Hexamethoxyflavone | 8.3 | 37.528 |
|  | **Total** | **93.37 %** |  |
